# Genres and typologies of standard paediatric service public funding model provisions for speech-language pathology management: A scoping review^☆^

**DOI:** 10.1016/j.hpopen.2026.100173

**Published:** 2026-05-19

**Authors:** T. Nickless, B. Davidson, L. Gold, R. Dowell

**Affiliations:** a Department of Audiology & Speech Pathology, The University of Melbourne, Australia; b Word By Mouth Speech Pathology, Melbourne, Australia; c Deakin Health Economics, School of Health & Social Development, Deakin University, Australia; d The Royal Victorian Eye & Ear Hospital, Melbourne, Australia

**Keywords:** Public funding models, Funding mechanisms, Private practice, Speech-language pathology

## Abstract

**Background:**

Successive Australian healthcare policies promoting privatisation have increased demand for independent speech-language pathology (SLP) services. This growth has intensified pressure on healthcare funding mechanisms and highlighted confusion in the literature between health financing systems and healthcare funding provisions at the funding model level. In response, public funding arrangements have increasingly supported access to private SLP services for Australian children and young people. This scoping review aimed to identify and describe current public funding models (PFMs) used to access standard paediatric SLP funding through Australian independent SLP providers.

**Method:**

Scoping review methodology was undertaken and incorporated search terms synonymous with funding mechanisms, population-based, block and individualised funding.

**Result:**

Iterative searching and screening of three health and two scholarly business databases unveiled eighty-six (n = 86) articles, revealing six genres of PFMs and nine associated typologies. The review identified that seven (n = 7) contemporary PFMs were used to access Australian independent SLP services.

**Conclusion:**

Health funding and health financing serve different functions within healthcare systems. Compared with the extensive literature on health financing, research on health funding remains limited. A key contribution of this scoping review was the identification of themes and the systematic classification of PFMs by genre and typology, providing a blueprint for understanding past, current, and future PFMs. Furthermore, mapping Australian independent SLP services across funding models and typologies highlighted the diversity of public funding provisions within Australia. Future research should examine how policy architects can balance increasing demand, integration of the latest scientific evidence-base, and equitable access to SLP services.

## Highlights

• *Clarification of health funding versus health financing:* This article provides conceptual clarity by distinguishing health financing from health funding – terms that are often inaccurately applied and used interchangeably within the Australian healthcare system. By defining their distinct functions and associated actors, it reduces ambiguity and establishes a stronger, more coherent foundation for policy design, funding reform, and allied health service planning.
• *Systematic classification of public funding models (PFMs):* This scoping review identified seven contemporary PFMs, organised into six genres and nine typologies, providing the first structured framework to understand how public funding enables access to independent speech-language pathology (SLP) services in Australia.
• *Critical gap and future policy opportunity:* This study highlights a significant evidence gap in health funding research and presents a blueprint for policy architects to design efficient funding models that balance rising demand with equitable access to paediatric SLP services, with clear applicability across other allied health sectors.

## 1 Introduction

Funding services is critical in the management of individuals and families with chronic and complex healthcare needs. Without the allocation of adequate *funding,* individuals cannot access timely and appropriate healthcare services. Within an Australian context, the term *funding* is widely used within the healthcare industry and is often used interchangeably with principles of *health financing*. Whilst both concepts are interrelated, it is however necessary to make the distinction between *funding* and *financing* of healthcare services as both terms differ in their functions; there is much confusion of the two concepts in the literature [1]. Whilst there is an extensive body of literature on health financing at a system level, there is a notable gap regarding how specific funding models (FMs) (i.e., model-level funding provisions) shape access to independent paediatric speech-language pathology (SLP) services in Australia [2].

This paper outlines the approach, when compared with an international context, of public funding mechanisms allocated to funding arrangements within Australian independent SLP private practice settings for paediatric services. Compared to alternative approaches internationally, the allocation of public funding for use within the independent SLP sector in Australia provides access to critical and timely SLP services. Moreover, access to public funding options for children and young persons [3] with swallowing and communication difficulties (from this point forward, known as *the population*) greatly assists individuals and families in the engagement of crucial yet costly primary care services through independent providers.

**Fig. 1:**
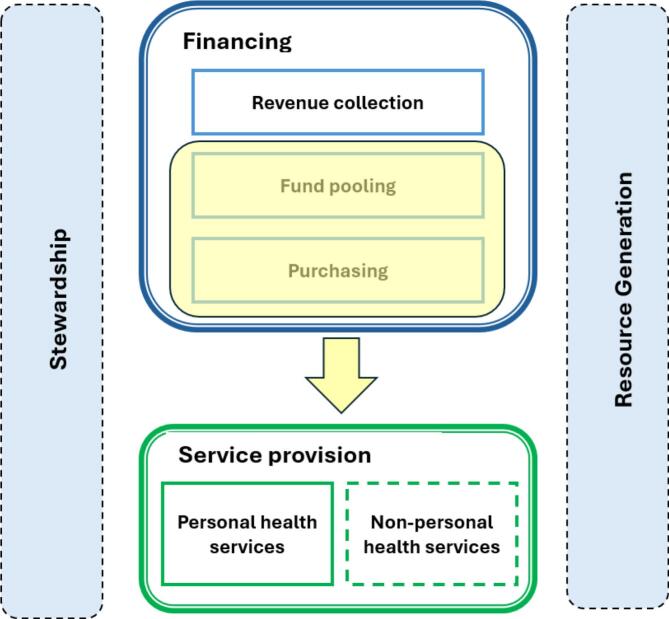
Functions of a healthcare system. Note: Adapted from Murray CJL, Frenk J. A framework for assessing the performance of health systems. Geneva: World Health Organization; 2000 [6]. Licensed under CC BY-NC-SA 3.0 IGO. This adaptation was not created by WHO; WHO is not responsible for its content or accuracy. The original edition remains the authoritative version. Accessed 2024 May 26.

The simplest definition is that *funding* is the act of allocating financial resources (i.e., money, effort, time) by an individual, organisation or government for a specific purpose or program [4]; whereas *financing* is the process of receiving capital or money to pay for a specific purpose or program [5]. For the purpose of this paper, *health funding* is defined as the process and system of transferring funds raised by the financing system to pay for the services rendered by health providers; that is, a *funding model* describes the mechanisms and provisions by which funds arrive at a service or organisation as the end-point of the *health financing* process [6], [7] and thereby acts as “*policies for distribution of benefits*”, i.e., *funding* [8, p.107]. Conversely*, health financing* is defined as the “*function of a health system concerned with the mobilization, accumulation and allocation of money to cover the health needs of the people, individually and collectively, in the health system… the purpose of health financing is to make funding available, as well as to set the right financial incentives to providers, to ensure that all individuals have access to effective public-health and personal healthcare*” [9, p.72]. *Health financing* is, therefore, the process of raising revenues to create a funding pool with which to pay for services through instruments such as taxation, statutory insurances/levies, voluntary insurance premia and out-of-pocket payments [10], [11]. Furthermore, when health professionals refer to *funding,* they are often referring to the *bucket of money* that is raised by revenues, taxes, levies (i.e., *health financing*) rather than the *funding* flows and transfer of payments in order for individuals to access healthcare (i.e., *health funding*). Murray and Frenk [6] identified four functions of a health system (see Fig. 1): stewardship, financing, service provision, and resource generation. This paper will investigate and clarify the definition of the term *health funding* using elements identified in Fig. 1 (e.g., applying the yellow highlighted components of *health financing* such as *fund pooling* and *purchasing* together with *provision of services*).

It is important to clarify the functions of a health financing system and associated actors. Health financing arrangements are conceptualised through four interrelated core functions: (a) coverage and benefit entitlements; (b) the collection of funds; (c) pooling of financial resources; and (d) purchasing and remuneration of providers [10]. Firstly, *coverage and benefit entitlements* refers to who is eligible for publicly financed healthcare, which services are included, and the extent of cost coverage, thereby determining population coverage, service scope, cost-sharing arrangements, and financial protection to achieve universal access [10, pp.1–10]. Secondly, *collection of funds* refers to the mobilisation of prepaid financial resources for healthcare (e.g., by individuals, households, employees and employers/organisations) through taxation (direct and indirect), social security contributions, voluntary insurance premiums, and out-of-pocket payments [10, pp.11–22]. Thirdly, *pooling of financial resources* describes the aggregation of collected revenues into one or more funding pools to spread financial risk across individuals and population groups, enabling cross-subsidisation, equitable resource allocation, and protection against catastrophic health expenditure [10, pp.22–26]. Last, *purchasing and remuneration of providers* involves allocating pooled funds to providers through contractual and payment arrangements, shaping service delivery, efficiency, quality, and provider behaviour within the health system. Within the health financing framework, funds are collected by actors (e.g., federal/state governments and households), pooled and managed by payer agencies (e.g., health and education departments or insurers), and used to purchase services from providers (e.g., medical and allied-health professionals). Busse et al’s [10] framework is used to examine the Australian context for SLP and other services (see Table 1).

**Table 1:** Definitions of funding model genres, associated typologies, funding flows and examples.

**Funding model genre**	**Definition of funding model genre and associated typology**	**Australian examples**	**Australian Health Financing Context**				**International examples**
			**Revenue collection**	**Pooling / coverage**	**Purchasing agency**	**Provider payment mechanisms**	
I. **Conventional healthcare financing mechanisms/ funding sources**	*Conventional healthcare* funding refers to the provision of funding via government universal or semi-universal healthcare support which is raised by taxes, other insurance measures (e.g., private health), and out-of-pocket payments, where patients pay directly for healthcare services without third-party support, government funding or other insurance measures.^a^	Hospital funding, national health insurance models (some elements of Medicare), private contributions to healthcare.^a^	General taxation; levies; out-of-pocket/self funded	Government budget allocations	Federal and State Governments	Multiple Payer; Fee-for-service; direct payment	United Kingdom: • The National Health Service (NHS) uses the Beveridge model; is tax funded and universal.^m^

II. **Insurance Schemes** ***Health Insurance***	*Health insurance* is a publicly funded universal healthcare insurance scheme where healthcare financing is designed to meet the cost of all or most healthcare needs from a publicly managed fund. In Australia this is operated by the nation’s social security agency, Services Australia.^b^	Medicare Benefits Scheme: • Item 10970: Chronic Disease Management Plan • Items 82005 & 82020: Helping Children with Autism & Better Start (Assessment and Treatment items) • Item 81360: Allied Health Services for Aboriginal and Torres Strait Islander Descent	General taxation; Medicare Levy	National statutory insurance	Services Australia (Department of Health and Ageing; Medicare)	Single Payer; Medicare Benefits Scheme	Germany: • Gesetzliche Krankenversicherung (Statutory Health Insurance; covers 90 % of the population).^n^ France: • Assurance Maladie component of the Sécurité Sociale.^o^
***Social Insurance***	*Social insurance* is a publicly funded government program where financial support is provided to individuals based on social fairness needs and equity (e.g., disability). In Australia, the National Disability Insurance Agency provides economic security and welfare through principles of fair burden sharing.^c^	National Disability Insurance Scheme: • Early Childhood: Age < 9 years Ages 9–65 years	Compulsory levy, funded by increase of 0.5 % to Medicare Levy	Social statutory insurance	National Disability Insurance Agency (NDIA)	Single Payer; Individual allocation to NDIA budgets: Capacity Building and Support	Norway: • National Insurance Scheme (Folketrygden).^p^
***Third-party insurance***	*Third-party insurance* is a form of liability insurance where protection is provided for an injury regardless of fault.^d^	SLP treatment related to accident injuries though: • Transport Accident Commission (Victoria) • SafeWork (New South Wales) • Department of Veterans’ Affairs (Cth)	Levies; voluntary insurance premia	Compensation pool	Insurers (e.g., Transport Accident Commission [Victoria])	Multiple Payer; Negotiated fees	Japan: • Workers’ Accident Compensation Insurance (Rōsai Hoken).^q^
III. **Education funding**	*Education equity adjustment* (i.e., loadings) for students with additional needs is a targeted supplementary funding program to support inclusion for defined populations of eligible students with disabilities within educational settings.^e^	Students with Disabilities, Independent Schools Victoria • Speech, Fluency, Language, Voice, Alternative Communication	General taxation	Education budget allocation	Education systems: (State Departments of Education; Independent Schools; Catholic Education)	Multiple Payer; Program specific funding	Canada: • Program Unit Funding (PUF) used by schools to access private allied-health for preschoolers with significant needs.^r^
IV. **Population-based & patient-focused funding** ***Population-based funding***	*Population-based funding* seeks to equitably distribute public funding based on the needs and characteristics of a population (i.e., age, socioeconomic status, ethnicity, geography, and disease status). Four values guide population-based funding mechanisms: transparency, accountability and integrity, equity and innovation.^g^	• Aboriginal Community Controlled Health Services, Non-Government organisations that employ GPs on a sessional basis.^h^	General taxation	Population pool (geographic region, e.g., health district; cultural group, e.g., First Nations community)	Commissioners	Multiple Payer; Block funding	United Kingdom (England): • Early Language Support For Every Child (ELSEC) program, but delivered by public SLPs.^s^
***Patient-focused funding***	*Patient-focused funding* is where the provision of funding is used to provide incentives and supports to providers to improve appropriateness, quality, and efficiencies in care for patients. ^h^	• Pay-for-performance (payment to providers or practices based on the type and number of specific services provided) and fee-for service (providers bill for each item of service they provide).^h^	General taxation	Program pool (indigenous health initiatives; grants, e.g., Corrections Victoria Dyslexia Health Program)	Funders	Multiple Payer; Pay-for-performance; Fee-for-service	France • Assurance Maladie by ‘contrats incitatifs’ conventions/ incentive contracts.^t^
V. **Performance driven**	*Performance-driven funding* seeks to improve healthcare through the provision of financial incentives based on the verified quality of outputs produced, modified by quality indicators.^i^	• Activity-based funding where funding is provided based on expected cost for clinically defined episodes of care; and casemix funding which adequately accounts for case complexity of patients and equitable risk sharing between funders and providers.^j^	General taxation	Performance pool (e.g., Hospital performance payments; quality improvement incentive programs)	Funders	Multiple Payer; Activity based funding; casemix	New Zealand: • Accident Compensation Corporation (ACC)’s Allied Health Services Contract where funding is based on timely access, accurate diagnosis/assessment, clinically appropriate and evidence-based treatment, education for clients to achieve ‘outcomes’ as agreed.^u^
VI. **Value-based**	*Value-based funding* strives to change healthcare behaviours by improving value through financial incentives offered to providers. The underlying premise is repositioning financial risk from funders to providers. Through incentives, providers are supported to improve outcomes that matter to patients and minimise operating expenses associated with the delivery of healthcare services.^k^	• Collaborative commissioning in New South Wales provides incentives for the delivery of value-based healthcare through a range of settings, including community and tertiary settings.^l^	Blended funding including federal and state budget allocations	Integrated pool	Commissioning organisations	Multiple Payer; Bundled; outcome driven	Scotland: • A general ‘Value Based Health & Care’ strategy which mentions allied-health professionals and multidisciplinary teams.^v^

Note. The framework developed by Busse et al. [10] was used to outline the functions of the health financing system (e.g., revenue collection, pooling, purchasing agency, provider payment mechanisms) and the roles of key actors; General taxation refers to direct and indirect taxation: (a) federal taxation e.g., income tax; Goods and Services Tax (GST); company tax; Fringe Benefits Tax (FBT); excise and duties; (b) state taxation e.g., payroll tax; land tax; stamp duty; ^a^[36], [37]; ^b^[38], [39], [40]; ^c^[41], [42]; ^d^[43]; ^e^[44], [45]; ^f^[46]; ^g^[47]; ^h^[48]; ^i^[49], [50]; ^j^[51]; ^k^[52]; ^l^[53]; ^m^[54]; ^n^[55]; ^o^[56]; ^p^[57]; ^q^[58]; ^r^[59]; ^s^[60]; ^t^[61]; ^u^[62]; ^v^[63]; Cth = Commonwealth.

The focus of this paper is with public funding provisions that provide access to SLP service for *the population* within independent settings in Australia. In Australia, speech-language pathologists are university trained in the management of swallowing and communication difficulties (e.g., swallowing/feeding, speech, language, literacy, voice, fluency, augmentative and alternative communication). Two key rationales underpin the focus on children and young persons in this review. First, they represent the primary caseload for Australian speech-language pathologists working in independent practice: in August 2024, 55 % of Speech Pathology Australia members practised within independent settings, and 83 % worked with children and young persons across all settings [12]. Second, major PFMs indicated high use by this group. Between January and March 2024, 89 % of Medicare Chronic Disease Management Plan SLP services were provided to children aged 0–14 years [13]. Over the same period, 52 % of National Disability Insurance Scheme (NDIS) participants receiving allied-health supports were aged 0–18 years [14]. Therefore, given the high incidence of children and young persons accessing these popular PFMs, it was considered appropriate to investigate PFMs specific to *this population*. The aims of this paper are to: (1) undertake a scoping review of peer-reviewed and grey literature in order to identify Australian PFMs and their arrangements; (2) identify which PFMs are available for use by children and young persons with communication and swallowing difficulties and their families in accessing Australian independent SLP practice; (3) clarify the definition of a *healthcare funding model*; and (4) develop a proposed classification framework that systematically articulates the conceptual architecture of a healthcare FM.

## 2 Materials and methods

### 2.1 Methodology

To identify relevant funding sources, associated mechanisms, structures, and models that assist individuals with communication and swallowing difficulties in accessing services, a scoping review methodology described by Arksey & O’Malley [15] was applied to synthesise and report on the breadth of literature. Thus, this scoping review included five progressive stages: (1) confirming the research question; (2) identification of relevant studies; (3) defining the study selection criteria; (4) synthesising data from the literature; and (5) collating, summarising, reporting, and interpreting the results. Given this literature review did not involve human participants, ethical approval was not required.

### 2.2 Inclusion criteria and definitions

Given the limited evidence within the literature pertaining to the umbrella term *funding models*, the inclusion criteria were broadened to include literature that addressed overarching *funding* mechanisms relevant to all allied-health professions within an international context (i.e., structures and mechanisms such as activity-based funding, population-based funding, blended funding, block funding, individualised funding), were published in English and relevant to the Australian context, and pertained to jurisdictions with Beveridge or Bismarck financing arrangements. To capture Australian funding initiatives, the search included the names of specific funding programs (e.g., Chronic Disease Management Plan [or its former title, Enhanced Primary Care Plan], National Disability Insurance Scheme, and variants such as *EPC* or *NDIS*). By relaxing the inclusion criteria, it was possible to dissect those funding frameworks available to SLP within health, education, disability, and community sectors. Searches were conducted with date restrictions applied up to March 2025. Literature addressing cost structures, cost analyses, and service delivery in relation to funding design were omitted. Studies were excluded where the focus was limited to research funding disclosures, adult-only populations, low- and middle-income country contexts, or private health insurance, thereby maintaining analytic coherence with publicly funded system design and policy relevance. This provided an opportunity for review and discussion, including (1) a snapshot of PFMs and their structures, (2) arrangement of PFMs according to their genre and typology, and (3) examples of PFMs employed to deliver SLP services.

Given the training of speech-language pathologists within the university sector and the manner in which speech-language pathologists conduct their professional services in Australia, speech-language pathologists are defined as *health professionals* who provide *healthcare* services within and across various sectors including health, disability, education and community settings. Due to the breadth of this topic, independent private practice has been included as part of the community sector.

### 2.3 Search strategies

The search strategies were designed with the support of an experienced librarian. Three health databases (e.g., CINAHL – The Cumulative Index to Nursing and Allied Health Literature, Medline Ovid, PubMed) and two business databases (e.g., Business Source Complete and Econlit) together with web-based search engines (e.g., Google and Google Scholar) were searched using the terms *funding models, traditional funding, population-based funding, activity-based funding, block funding, individualised funding, value-based funding*. Database searches using the abovementioned terms and Boolean phrases (including truncations; e.g., fund*, pathology*, individuali$ed) were used in combination with the primary term speech-language pathology or a variant term (e.g., speech therapy) and/or children or a variant term (e.g., paediatric). In addition, specific FMs were also included in the search (e.g., Chronic Disease Management Plan; Helping Children with Autism, Better Start; National Disability Insurance Schemes or variant NDIS). Refer to Supplementary Material I (Literature search strategy), for complete list of Boolean phrases, primary and variant terms. The search process did not incorporate manual searching of journals or scanning of footnote entries.

### 2.4 Limitations and search expansion

The nature of the original question (i.e., SLP funding of children and young persons within an Australian context) posed the greatest challenge to the initial literature search. A pool of reliable studies to draw concise and purposeful conclusions was unavailable, initially resulting in an empty review [16]. Other studies specific to SLP briefly discussed themes relating to service delivery and cost to access SLP [17], [18], [19], [20], [21], [22]; however, there were no studies that discussed in detail the *funding provisions* available for access to SLP services. Three studies explored in detail the economic modelling (including cost savings) of SLP services within criminal justice systems [23] and the economic impact of childhood developmental language disorder [24], [25]. Nonetheless, the foci of these studies were on economic modelling/impact; not *funding provisions*. Two education-sector studies [26], [27] appeared relevant to SLP funding but were excluded after full review, as they focused on: (1) teacher and SLP perceptions of service need and preferences; and (2) access to support with limited discussion of funding criteria. Many studies in the disability and mental health sectors also discussed general health financing principles (e.g., [28], [29], [30], [31]), but were excluded because they did not specifically examine FMs relevant to SLP and therefore did not meet the inclusion criteria. One of the primary search terms *funding* also identified a plethora of articles; the content of these articles, however, did not include any discussion(s) that related to healthcare funding or FMs specific to SLP or allied-health, but rather medical FMs involving diagnosis related groups [32], [33], [34], [35]. In addition, publications that only identified funding of research within the declaration of interest sections were removed.

### 2.5 Data analysis

Data were extracted and then analysed according to six FM genres defined in Table 1. This literature review applied the following definitions: (a) *funding model genre*, which considered the classification of PFMs according to defined characteristics including the structure, architecture, and aspiration of the FM (e.g., population-based and patient-focussed versus performance-driven PFMs); and (b) *funding model typology*, that differentiated the systematic classification of PFMs as a consequence of their distinct mechanisms and overall function and purpose (e.g., health/social/third-party insurance, education funding, population-based funding, patient-focussed funding). When analysing the dataset, quantitative synthesis was not performed; instead, findings were examined descriptively to capture conceptual and contextual patterns across FM genre and typology.

### 2.6 Results

**Fig. 2:**
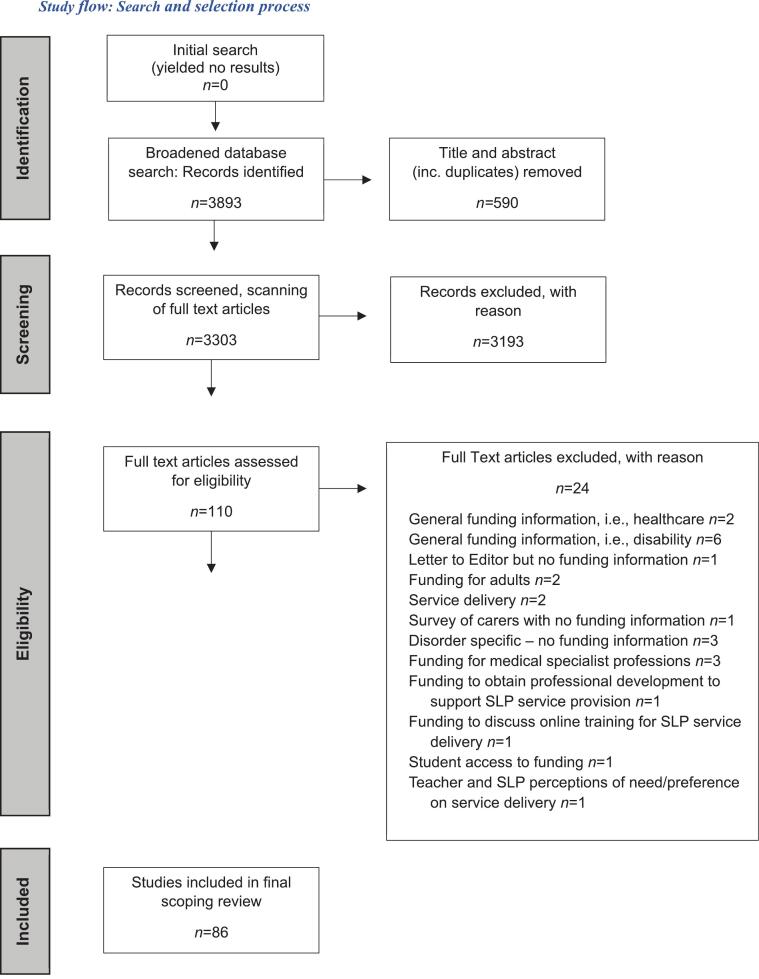
Study flow: Search and selection process.

Through broadening the search criteria, 3893 documents were identified with 590 duplicates. Approximately 96.7 % (n = 3193) weeting above exclusion criteria) through a process of screening (i.e., title and abstract review) which left 110 articles (n = 110) available for eligibility through full text analysis and review. Twenty-four articles (n = 24) were excluded for the reasons provided in Fig. 2. Eighty-six papers (n = 86) met the inclusion criteria. Fig. 2 illustrates the study flow using a search and selection process. Included studies are numbered and reviewed in Table 2. To facilitate analysis in the results and discussion sections, studies are referenced according to the study number identified in Table 2.

**Table 2:** Key components of the 86 included studies

**Study number(#)**	**Referencenumber[#]**	**Date range of publication**	**Country**	**Aim of study**	**Study design**	**Level of evidence^a^**	**Aspects of funding discussed**	**Funding modelgenre^b^**	**Funding model typology^c^**
(1)	[64]	2010–2014	Australia	To assess whether the components of the Healthy Kids Check, a preschool screening check recently added to the Australian Government’s Enhanced Primary Care Program, are supported by evidence-based guidelines or reviews.	Systematic Review	1	Funding model and evidence did not align. Guidelines were often inconsistent in their recommendations. Most of the components of the Healthy Kids Check were not supported by evidence-based guidelines relevant to the primary care setting, though several consensus-based guidelines are supportive.	II & IV	Health
(2)	[65]	Before 2005	Australia	To develop a capitation funding model for cystic fibrosis in an Australian health maintenance organisation.	Cohort study (n=173)	5	Discussion of blended capitation funding and fee-for-service payment systems as an alternative to casemix funding	II	Health
(3)	[66]	2015–2019	Australia	To describe service choices, costs, out-of-pocket expenses, and the impact on families.	Survey design using participants from one early intervention facility (n=23).	2	Families of young children with multiple disabilities select a wide range of services for their child, with consequential out-of-pocket expenses.	II	Disability
(4)	[67]	Before 2005	Australia	To provide review of three approaches to funding.	Narrative Review	5	Review of historical funding, population-based funding and output-based funding.	IV	Health
(5)	[68]	2005–2009	Australia	To measure empowerment levels of those people in receipt of direct funding.	Survey design using random sample from database of parents/carers of people with intellectual disabilities. Participants (n=424).	2	Comparative analysis of families with direct funding versus those without funding. Measures included four dimensions of empowerment — meaning, competence, self-determination, and impact of direct funding.	II	Disability
(6)	[10]	2005-2009	Multinational	To inform the policy debate related to health financing in high income countries.	Discussion paper comparing high income countries (n=25)	5	Review based on a conceptual framework of health financing functions. Comparative evaluation of twenty-five high income countries based on healthcare financing mechanisms; also theoretical discussion of funding flows and various functions of health financing.^d^	II	Health
(7)	[69]	Before 2005	Australia	To outline the benefits of casemix funding from allied-health perspectives.	Commentary	5	Discussion of casemix funding and the opportunity for allied-health to review their approaches for patient care, reduce organisational costs, maximise reimbursements under funding rules, and improve quality care.	V	Health
(8)	[70]	2005–2009	Australia	To describe private practitioner dieticians’ experience of the Enhanced Primary Care program funded under the national health insurance.	Qualitative semi-structured interviews (n=15)	3	Thematic analysis identified five themes involving challenges with the implementation of the funding model: referral issues, client preparedness, annual number of consultation limit, impact of financial cost on client, and non-reimbursed administration costs.	II & IV	Health
(9)	[71]	2010–2014	Australia	To critically examine utilisation of the 13 allied-health services provided through Medicare Chronic Disease Management program and related General Practitioner care planning initiatives.	Retrospective study	2	Wide variation was apparent in per capita utilisation of allied-health services by State or Territory; some with far less than average national use and others with high use. Authors concluded that the funding model fosters inequality of accessibility for patients. The authors also proposed that a review of funding arrangements is warranted.	II & IV	Health
(10)	[72]	2015–2019	New Zealand	To gain a better understanding of how population-based funding formula is portrayed in the news media and of perceptions of funding allocations across the country.	Qualitative thematic analysis	3	Thematic analysis identifying dissatisfaction with population-based funding formula across geographic regions.	IV	Health
(11)	[73]	2020–2024	Multinational	To identify international funding models and to summarise evidence underpinningtheir efficacy in hospital systems.	Systematic review (n=6)	4	Review of hospital funding in 15 Organisation for Economic Cooperation and Development countries. While some models adjusted for disadvantages and ethnicity factors, the review did not identify literature specific to health-systems that adjusted funding allocation for social determinants such as health literacy levels.	IV	Health
(12)	[8]	Before 2005	United States	To review five ethically based healthcare funding models that are currently used to justify funding choices.	Narrative review	5	Review of models of healthcare funding.	II	Health
(13)	[52]	2020–2024	Australia	To provide a roadmap for the transition to a value-based healthcare funding model within an Australian context.	White paper	5	A report outlining benefits of transitioning to value-based payments for the Australian healthcare system	VI	Health
(14)	[74]	2010–2014	Australia	To identify benefits and barriers for rural and remote service users and service providers using individualised funding models.	Focus groups and interviews conducted with service providers and carers. Participants: services providers (n=97); carers (n=78).	3	Comparison of international and Australian experience of individualised funding and opportunities for self-determination. Specific discussion of Helping Children with Autism, Better Start Initiative and National Disability Insurance Scheme. Discussion of implications for families in rural and remote locations.	II & IV	Disability
(15)	[75]	Before 2005	Australia	To outline and compare the different funding models used within the Australian health system.	Narrative review	5	Discussion of casemix funding arrangements investigating funding models and comparison of Australian state-based health-systems.	V	Health
(16)	[76]	2015-2019	Multinational	To outline four areas where Canada could potentially learn from Australia in expanding health insurance.	Commentary	5	Commentary outlining historical funding arrangements from the Australian experience including the role of allied-health professionals using ‘care coordination’ programs and the efficiencies used in the Australian hospital system. Author provides four learning opportunities for Canada from the Australian experience.	II, IV, & V	Health
(17)	[77]	Before 2005	New Zealand	To provide justifications for use of population-based funding mechanisms in New Zealand	Narrative Review	5	Strengths and weaknesses specific to the population-based funding formula.	IV	Health
(18)	[78]	Before 2005	Australia	To develop a classification system that could be used to fund therapy services for children from 5 to 18 years old with disabilities	Observational case study	2	Discussion of funding model has the potential to be used to purchase services on a fairer basis than traditional, historical funding methods have allowed.	IV	Disability
(19)	[79]	2020–2024	Australia	To estimate the burden of disease and evaluate which factors affect health care resourcing us in your children with cerebral palsy.	Prospective longitudinal study (n=222)	2	Discussion of Better Start for Children with Disability Initiative and National Disability Insurance Scheme and levels of funding for preschoolers with cerebral palsy.	II & IV	Disability
(20)	[80]	2015–2019	Australia	To explore parental perspectives on early intervention messages.	Qualitative semi-structured interviews (n=14)	3	Findings suggest parents were acutely aware of the importance of early intervention. Discussion on funding models and parental decision making regarding which intervention approaches to access for their children with autism spectrum disorder.	II	Disability
(21)	[81]	2010–2014	Australia	To examine the effectiveness of individual funding of disability support and to inform policy to improve the provision of disability support.	Government report	5	Person centred individualised funding and impact on social and economic inclusion.	II	Disability
(22)	[82]	2015–2019	New Zealand	To review the effect of the population-based funding scheme introduced in New Zealand as a component of the New Zealand Primary Health Care Strategy.	Narrative review	5	Review outlining the introduction a national, comprehensive program to improve access and reduce health disparities. Successful implementation of a complex set of interventions to address inequity has challenges.	IV	Health
(23)	[83]	2005–2009	Australia	To examine the discretionary practices adopted by AHP in response to the realities at the policy-practice interface and situates the discussion within a description of their experiences with Enhance Primary Care initiative.	Qualitative semi-structured interviews (n=15)	3	Implications of restriction on the number of subsidised sessions was not conducive to providing an evidence-based allied-health service to patients with complex care needs. Discussion of dilemmas felt by allied-health professionals in quality patient care versus business viability business.	II	Health
(24)	[84]	2005–2009	Australia	To provide an overview of the policy induced dilemmas for allied-health professionals.	Commentary	5	Discussion on the Enhanced Primary Care funding initiative and the initiatives’ focus on multidisciplinary care. Authors highlight that the number of funded treatments is far less than standard clinic practice indicates which may compromise clinical outcomes and give rise to socioeconomic inequalities due to a patient’s ability to pay.	II & IV	Health
(25)	[85]	2020–2024	Australia	To explore how paediatric speech-language pathologists seek to involve parents in speech and/or language intervention funded by the National Disability Insurance Scheme.	Thesis	2	Thesis illuminated that the National Disability Insurance Scheme facilitated parent involvement in decision making for speech- language pathology intervention.	II	Disability
(26)	[17]	2015–2019	Australia	To examine the issues involved in the delivery of therapy services to people with disabilities in a rural and remote area to develop, implement and evaluate new sustainable models of service delivery.	Cross-sectional study using survey instrument with carers (n=166)	3	Discussion of limitations of access to service using direct and individualised funding in rural and remote areas (e.g., lack of choice, waiting times and availability of specialist disability services in rural areas).	II & IV	Disability
(27)	[86]	2020–2024	Australia	To describe the process, including stakeholder co-design, and lessons learned from the development of a value-based framework	Case-study	3	Outline of a value-based healthcare framework used in the Queensland public allied-health service.	VI	Health
(28)	[87]	Before 2005	United Kingdom	To investigate how far individuals with disabilities use direct payments to cross the health/social divide, and to describe the potential opportunities and barriers for extending this further.	Qualitative interviews with direct payment users (n=44).	3	A study of people with disabilities receiving direct payments who were able to purchase assistance in ways that traverse between “health” and “social” services.	II	Disability
(29)	[88]	2010–2014	Australia	To evaluate new pathways to access allied-health services introduced by the Enhanced Primary Care/Chronic Disease Management initiative that may both increase or decrease equity to and efficiency in access.	Qualitative semi-structured interviews with allied-health practitioners (n=15).	3	Discussion that the funding provisions of Enhanced Primary Care/Chronic Disease Management initiative appeared to address two key barriers of access to allied-health services: costs to access by patient, and patient awareness of benefits. However, gap payments exist which impact economically disadvantaged patients from attending.	II & IV	Health
(30)	[89]	2015–2019	Australia	To examine the perspectives of parents and carers of disabled children in one NDIS trial site.	Mixed methods comprising of survey (n=75) and semi- structured interviews (n=34).	2	Discussion of management of funding. Results illustrated that there was low priority from participants on controlling and managing funding packages for their child. However, coordination, flexibility, choice, and self-directed funding were seen as features of effective support.	II	Disability
(31)	[90]	2005–2009	United States and Canada	To provide a review of individualised funding arrangements in North America	Commentary	5	Individualised planning and direct funding have developed into a new model of disability supports for citizenship and inclusion.	II	Disability
(32)	[91]	2020–2024	Australia	To provide an overview of Australia’s progression to a value-based funding model and healthcare system.	White paper	5	Provision of examples of implementation barriers and initiatives relating to value-based funding and healthcare.	VI	Health
(33)	[92]	2015–2019	Australia	To explore private practice dietitians’ perceptions of the impact of the Australian Chronic Disease Management program on the conduct of their private practice, and the care provided to patients.	Qualitative semi-structured interviews (n=25)	3	Discussion of challenges faced by dieticians in using the Chronic Disease Management program (e.g., pressure from doctors and patients to keep fees low due to partial rebate).	II & IV	Health
(34)	[93]	2020–2024	Australia	To describe the understanding, experiences, and expectations of families living in rural and remote Australia regarding core concepts relating to disability service provision, including person-centred practice, family-centred practice, transdisciplinary practice, choice, control, inclusion, and equity, with a view to presenting a more coherent set of solutions and preferences for achieving choice and control.	Qualitative semi-structured interviews (n=13)	3	Findings reflect the individualised funding arrangements of NDIS. Thematic analysis revealed the following themes: (a) Relevance to the child; (b) Missing out on choice and control; (c) Inclusion versus isolation; and (d) Service provider skills and practices.	II & IV	Disability
(35)	[94]	2005–2009	Australia	To provide an overview of current chronic disease self-management support within Australia, with a specific emphasis on interactions between patients, health care professionals, and policy.	Narrative review	5	The authors conclude that a systematic approach that facilitates integration and enhances the interactions between patients and providers is an essential part of the funding model.	II & IV	Health
(36)	[95]	2020–2024	Australia	To investigate parents’ experiences and perceptions of finding and obtaining SLP services for children with communication disorders across Australia.	Survey design (n=107)	2	Identification of persistent barriers in accessing SLP services (e.g., availability, affordability, and geographical location). Families from rural/remote regions faced additional difficulty accessing services due to the ongoing shortage of services in these regions.	IV	Health
(37)	[53]	2020–2024	Australia	To provide an overview of regional funding models that support value-based care.	Opinion	5	Discussion of collaborative commissioning and the delivery of value-based care and associated funding models in New South Wales.	VI	Health
(38)	[96]	Before 2005	Canada	To outline the advantages and disadvantages of population needs-based funding model.	Thesis	2	Doctoral research on population needs-based funding for healthcare in British Columbia.	IV	Health
(39)	[97]	2010–2014	Australia	To provide a comparative analysis of the Chronic Dental Disease Scheme and the Allied Health Profession program as they related to greater Enhanced Primary Care.	Retrospective study	2	Comparative analysis suggests significant differences in costs, nature of treatment, and the manner of remuneration between dentistry and the AHPs. Discussion of (a) fee for service implications for Chronic Dental Disease Scheme, and (b) the amount of treatment deemed necessary may be influenced by the level of subsidy.	II & IV	Health
(40)	[98]	2010–2014	Multinational	To review social participation outcomes identified in discrete studies of flexible funding programs across four countries.	Qualitative structured and semi-structured interviews conducted with people with a disability, families, support services, government administrators, and researchers from 2005 to 2008 (n=56).	3	Investigation of flexible funding programs across four countries.	II	Disability
(41)	[99]	2010–2014	Australia	Two objectives: • To evaluate the effectiveness of the program’s implementation strategies and the outcomes achieved. • To present a case study and analyse the findings using four system levels to identify what factors were effective in the program’s implementation.	A qualitative case design using observation and interviews with program participants and managers (n=11) over a four-year period from 2003 to 2007.	3	Proposal of a framework for the introduction and implementation of individual funding arrangements based on the analysis of data collected in a qualitative case study conducted in an Australian not-for-profit disability agency.	II	Disability
(42)	[100]	2015–2019	Australia	To explore the factors that facilitated or hindered the implementation of individualised funding programs in Western Australia.	Exploratory case study consisting of in-depth interviews with key people involved in developing and implementing individualised funding in Western Australia (n=11).	3	Examination of whether individual funding packages achieved their objectives in Western Australia, where they had been the primary mechanism of disability support for over 25 years.	II	Disability
(43)	[101]	2020–2024	Australia	To discuss the advantages of dynamic ‘changing needs’ planning models, compared to ‘constant use’ planning models and consider a framework that integrates population needs directly into health service planning.	Retrospective study using representative sample from private households, aged 15 year and above (n=>17,000)	2	Discussion specific to needs-based funding. Conclusion that ‘constant-use’ planning models based on expected future numbers of specific demographic groups applied current levels of service without any consideration given to changing age-specific needs for healthcare, resulting in inefficient resource planning.	IV	Health
(44)	[102]	Before 2005	Canada	Three objectives: • To identify themes and lessons learned from several existing individualised funding projects in Canada and around the world. • To identify themes and lessons learned from research, literature, and government documents related to individualised funding. • To move the individualised funding agenda forward for individuals with disabilities.	Case study descriptions using a cross-site analysis.	3	Cross-site analysis of individualised and direct funding across Canada, United States, and Australia.	II	Disability
(45)	[103]	2020–2024	Australia	To better understand the experiences of early intervention providers who support young children on the autism spectrum, and their knowledge and values about evidence-based practices.	Qualitative semi-structure interviews (n=15)	3	Discussion of funding provisions to support families and individuals with autism spectrum disorder through National Disability Insurance Scheme and Helping Children with Autism initiatives.	II	Disability
(46)	[104]	2010–2014	Australia	To provide overview of casemix funding in healthcare and assessing casemix performance.	Narrative review	5	Discussion of World Health Organisation classification system to improve casemix funding.	V	Health
(47)	[105]	2020-2024	Australia	To gather rich, detailed information on complex needs in veteran families, and explore service providers’ and families’ experiences of accessing and navigating the veterans’ support system.	Qualitative semi-structured focus groups (n=18)	3	Service providers and families found the funding model and veterans’ support system challenging to access and navigate.	II	Third party
(48)	[106]	2015–2019	Australia	To explore, from administrators’ viewpoint, the impact on agencies and implication for practice.	Qualitative focus groups and interviews with administrators from two agencies involved in the NDIS roll out (n=6).	3	Investigated “user-pay” system. Findings suggest that the long-term financial viability of community agencies is at risk.	II	Disability
(49)	[107]	2015–2019	Australia	To describe the impact that self-determination of funding has on the therapeutic process.	Narrative review	5	Discussed self-determination of funding and the impact that this funding scheme may have on therapeutic approaches.	II	Disability
(50)	[108]	2005–2009	New Zealand	To provide mathematical models on population-based funding formulae.	Commentary	5	For equity and effectiveness, population-based funding needs to be implemented with appropriate frameworks. Example of mathematical models with specific focus on Māori population and forensic psychiatric services.	IV	Health
(51)	[109]	2015–2019	New Zealand	Technical report for population-based funding formula	Government report	5	Outline of funding formula for population-based healthcare.	IV	Health
(52)	[110]	2005–2009	Australia	Four objectives were identified: • To develop or identify a model of client classification related to resource usage; • To develop case list ratios for all therapy professions; • To commence case list allocation and staffing based on client ratios; and • To evaluate, modify, and promulgate the funding model.	Mixed methods including retrospective data mapping and qualitative focus groups, and in-depth interview with clients, providers of service, and corporate service staff (n= not reported)	2	Development of a funding model for allocation of staff time which reflected the differing service demands, travel time, leave allowances and time for activities to develop the social environment for individuals with disabilities.	IV	Disability
(53)	[111]	Before 2005	Multinational	To provide commentary on the historical report of significance: The Beveridge Report.	Commentary	5	Review of the 1942 report Social insurance and allied service by Sir William Beveridge.	I	Health
(54)	[112]	2010–2014	United States	To learn about state health departments’ use of funding formulas for specific public-health activities.	Survey design using participants from state health departments (n=40)	3	Review of federal and state strategies specific to their use of public funding formulas; sources of funding; formula attributes formula development and assessment against government policy.	IV	Health
(55)	[49]	2015–2019	Multinational	To explore the application of blended funding models in primary health care	Narrative review	5	Evaluation of the consequences in implementing blended funding models within Australia, New Zealand, and Canada. Evaluation included the impact of these approaches on organisations, care delivery for chronic conditions and patient experience.	II	Health
(56)	[48]	2010–2014	Australia	To examine the funding models that impact on primary health care service delivery.	Narrative review	5	Review of funding typologies and examples within the Australian context.	II & IV	Health
(57)	[113]	2015–2019	Australia	To investigate and report on the experience of a group of Australian Allied Health practitioners with the Medicare Chronic Disease Management program.	Survey design (n=85)	3	Participants reported that patients referred under a funding model would require an average of 8–10 consults per annum to provide optimal benefits for patients from osteopathic treatment.	II & IV	Health
(58)	[114]	2005–2009	Australia	To provide an overview of the key themes focusing on the experienced benefits and challenges associated with consumer-directed care.	Qualitative longitudinal study using observation and semi-structured interviews between 2003–2008 with carers (n=12)	2	Description of experiences in the development and implementation of consumer-directed care and funding implications on service.	II	Disability
(59)	[35]	2010–2014	Multinational	To explore how activity-based funding affects quality of care, access to care, equity, hospital and total health care system costs, length of stay, and efficiency.	Systematic review and meta- analysis	1	Analysis of introduction of activity-based funding and impact on health-systems.	V	Health
(60)	[46]	2010–2014	Multinational	To explore key similarities and differences across seven funding formulae and contrast against common demographic and geographic features.	Comparative analysis of convenience sample (n=7)	2	Comparison of differences in the composition of health funding formulae across seven health-systems.	IV & V	Health
(61)	[36]	2010–2014	Multinational	To provide an overview of the historical development and structure of healthcare models,	Narrative review	5	Description of four basic healthcare models used worldwide.	I	Health
(62)	[115]	2015–2019	Multinational	To provide an overview of how the Australian Federal and State governments can increase financial sustainability and quality of healthcare through evolving value-based funding models.	White paper	5	Discussion of value-based funding models through the provision of international examples.	VI	Health
(63)	[37]	2020–2024	Multinational	To provide analysis of efficiencies in health-systems over a five-year timeframe.	Scoping review	5	Brief overview of how health-systems are funded through four basic models.	I	Health
(64)	[116]	2015–2019	Australia	To evaluate parents’ feedback regarding their experience in registering and accessing funding with the National Disability Insurance Scheme and communicating with the National Disability Insurance Agency.	Survey design (n=129)	3	Findings revealed: (a) 36 (85.7 %) parents reported having no difficulty with the NDIS registration process; (b) six parents (14.3 %) had difficulty with registration process; (c) 27 (64.3 %) reported having no difficulty accessing NDIS; (d) 11 (26.2 %) stated that it was difficult to access; and (e) four parents did not comment.Findings also revealed: (a) 26 parents (61.9 %) reported that it was easy to communicate with the NDIA; (b) 12 (28.6 %) found it difficult. Overall, 26 (61.9 %) parents were satisfied with the NDIS and NDIA, six (14.8 %) were unsatisfied and nine (21.4 %) were neutral.	II	Disability
(65)	[117]	2015–2019	Australia	To describe the establishment of the National Disability Insurance Scheme in Australia.	Commentary	5	Context and discussion about the historical funding of disability services. Discussion about individualised funding from a worldview perspective	II	Disability
(66)	[118]	2015–2019	Australia	To provide a detailed overview of the 16 core components of the headspace centre model.	Commentary	5	Outline of blended funding model for youth mental healthcare.	II	Health
(67)	[119]	2015–2019	Australia	To examine whether different types of case management can mitigate that risk by providing support when people have only small direct funding packages.	A mixed methods longitudinal approach using semi-structured interviews with adult participants (n=15), family carers/supporters (n=7), family members with children with disabilities (n=15), and service providers (n=13)	2	Self-directed, individualised support packages and the implications of managing without sufficient funds.	II	Disability
(68)	[120]	2015–2019	Multinational	To review empirical literature (since 2010) that evaluates the effect of value-based purchasing in health care.	Scoping review (n=80)	2	A review of financial incentives to improve the provision of value-based health care. Eighty studies of 44 schemes from 10 countries were reviewed. Findings revealed that there were no differences between studies conducted across 10 countries.	VI	Health
(69)	[121]	2010–2014	Australia	To test the proposition that systematic variation in funding outcomes is independent of funding arrangements and can, instead, be explained by incremental cost effectiveness and intervention or patient characteristics linked to community values or health maximisation.	Systematic review of interventions (n=74)	1	Provision of a list of attributes that help to explain the possibility of health interventions being rejected for funding. The paper also provided the level of funding allocated for intervention that was funded.	V	Health
(70)	[122]	2015–2019	Australia	To examine self-directed funding models for families and children with disabilities.	Systematic review identifying twelve (n=12) studies of interest.	1	Although the overall quality of studies had a low to moderate rating, findings revealed that self-directed funding provided flexibility, autonomy, and greater social participation.	II	Disability
(71)	[123]	2005–2009	Australia	To examine speech-language pathologists’ views about patient suitability for the Enhanced Primary Care Program, and their practices in discussing the funding scheme with patients.	Survey design (n=541)	3	Evaluation of patient suitability and investigation on which patients are referred to the Enhanced Primary Care Program through their General Practitioner.	II & IV	Health
(72)	[124]	2010–2014	Australia	To examine speech-language pathologists’ views and experiences under Enhanced Primary Care Program.	Survey design (n=541)	3	Findings concluded that education around eligibility, access, and reporting requirements was needed for clients, General Practitioners, and allied-health professionals.	II & IV	Health
(73)	[125]	2005–2009	Canada	To provide a detailed historical description of the development of Alberta’s population-based funding model.	Narrative review	5	Provision of historical context of introduction of population-based funding model in Alberta.	IV	Health
(74)	[126]	2005–2009	Australia	To assess the extent to which quasi-market reform has improved user outcomes	Qualitative study using semi-structure interviews with individuals with disabilities (n=31) and carers (n=32).	3	Participants reported that individualised funding arrangements had not delivered thebenefits of a quasi-market model. Findings reported challenges with increased consumer choice and efficiencies in service delivery due to lack of funding.	II	Disability
(75)	[127]	Before 2005	United Kingdom	To evaluate the implementation of direct payments in two Welsh local authorities.	Mixed methods using survey (n=88) and in-depth interviews with social workers (n=8).	2	Direct payments for individualised care allowed greater flexibility which facilitated participant benefits (e.g., improved self-esteem, increased control over life, deeper longer-lasting relationships, vocational and lifestyle opportunities).	II	Disability
(76)	[128]	2010–2014	Australia	To provide an overview of the state of acquired brain injury disability for Aboriginal and Torres Strait Islanders in remote and outer regional settings, and the present sets of barriers they face to obtaining quality care and effective interventions.	Narrative review	5	Discussion outlining the barriers facing Aboriginal and Torres Strait Islander people living with disability in outer regional and very remote communities, and associated challenges of individualised, self-directed funding provisions.	II & IV	Disability
(77)	[129]	2020–2024	United States	To define and explain the implementation of value-based healthcare as a strategic framework.	Commentary	5	In using the value-based framework from Porter and Teisberg (2006), this article provides an outline of value-based healthcare within the context of health outcomes and cost.	VI	Health
(78)	[130]	2005–2009	Australia	To present a model of fund allocation that provides a communication construct that addresses the needs of both policy makers and service providers.	Commentary	5	Presentation of a funding model that allows public funders and dental service providers to engage in funding agreements that both quantify and justify recommended treatments.	V	Health
(79)	[131]	2010–2014	Australia	To describe funding models used and compare the effects of funding models for remuneration on clinical activity and cost-effectiveness in outreach eye services in Australia.	Cross-sectional study using qualitative semi-structured interviews (n= not reported)	3	Discussion of the efficiency and costs of services specific to reimbursement to providers via funding models. Description of enablers and barriers for different funding models. Further exploration of funding models suitable to ensure isolated and disadvantaged areas prone to poor patient attendance are not neglected is recommended	II	Health
(80)	[51]	2010–2014	Multinational	Three objectives: • To provide an overview of the development of casemix in rehabilitation; • To describe key characteristics of some well-established casemix and payment models in operation around the world; and • To explore opportunities for future development arising from the lessons learned.	Narrative review	5	Review of casemix in rehabilitation and the challenges for healthcare systems worldwide. Discussion of successes and failures of casemix systems in the United States and Australia. Authors outline the importance for casemix funding models to cater for different healthcare cultures, and support treatment for patients with complex needs.	V	Health
(81)	[132]	2020–2024	Australia	To investigate collaborative partnerships between three stakeholders that often support students with autism spectrum disorder: teachers, parents, and allied-health professionals.	Survey design (n=129)	3	Discussion of inclusive education and education funding models to support children with autism spectrum disorder.	III	Education
(82)	[133]	2020–2024	Australia	To discuss the use of retrospective clinical audits in complex Allied Health interventions, specifically based on our experience of designing and trialling a clinical audit in one service delivery area of occupational therapy	Retrospective study	3	Implication of funding restrictions related to Helping Children with Autism funding initiative and National Disability Insurance Scheme (e.g., misinterpretation of professional reports)	II & IV	Disability
(83)	[134]	2020–2024	United States	To provide an outline for the introduction of a value-based funding model strategy, focusing on moving from traditional fee-for-service approaches to value-based funding models.	White paper	5	Discussions around the reasons for the implementation of a high-quality, efficient healthcare system that applies value-based funding models to achieve healthcare equity.	VI	Health
(84)	[135]	Before 2005	United Kingdom	To investigate the role parents are playing in direct payments provision for their child with intellectual disabilities.	Qualitative interviews with family carers (n=29)	3	Direct payments for individualised care.	II	Disability
(85)	[136]	2020–2024	Australia	To describe options for value-based payment reform and highlight two challenges critical for success: attributing financial risk fairly, and organisational structures.	Opinion	5	Discussion that ‘Fee for service’ is the dominant payment method in Australia, creating incentives to increase service volume, rewarding inputs rather than improvements in longer-term health outcomes. Authors suggested payment reform is needed to support the shift to value-based healthcare in Australia.	VI	Health
(86)	[137]	2010–2014	United Kingdom	To provide information on the resource allocation formula for distributing funding for health services in England to local clinical commissioning groups.	Government report	5	Discussion of the funding allocations using population-needs funding to Clinical Commissioning Groups.	IV	Health

Note. ^a^Levels of Evidence for Meaningfulness [138], [139]; ^b^I = conventional healthcare financing mechanisms/funding sources; II = insurance schemes; III = education funding; IV = population-based and patient-focused funding; V = performance driven funding; VI=value-based funding; ^c^adapted from [8]; ^d^[10, p.8] incorrectly reported that the Australian Medicare system did not cover allied-health services. Medicare, however, has provided health insurance rebates for allied-health services since 2004 (refer to [2]); Also refer to Table I in [140]; For evidence describing the Australian situation, disability refers to funding models prior to the introduction of the social insurance funding model in July 2013, Australia’s National Disability Insurance Scheme. Studies are presented in alphabetical order by first author surname.

### 2.7 Characteristics of evidence

Supplementary Material II (Characteristics of Evidence) summarises the characteristics of the 86 studies included in the scoping review. Identified studies were published between 1991 (study 4) and 2024 (studies 19, 27, 32, and 45), with 49 (*n* = 49, 56.9 %) published after 2010. Studies were conducted in a range of geographic regions, with most publications describing the Australian FM landscape (*n* = 57, 66.3 %). Publications identified two dominant FMs: (a) *health insurance models* (*n* = 53, 61.6 %); and (b) *disability and/or social insurance models* (*n* = 31, 36.0 %). Level of evidence for each study was determined using *Level of Evidence for Meaningfulness* [138], [139]. Studies included examples from all five levels of evidence, with the largest number of studies falling in the lowest ranking evidence category *Level 5 Expert Opinion* (*n* = 36, 41.9 %), closely followed by *Level 3 Single qualitative study* (*n* = 28, 32.6 %). There were five systematic reviews (studies 1, 11, 59, 69 and 70), four of which were rated as *Level 1* evidence (studies 1, 59, 69 and 70).

**Fig. 3:**
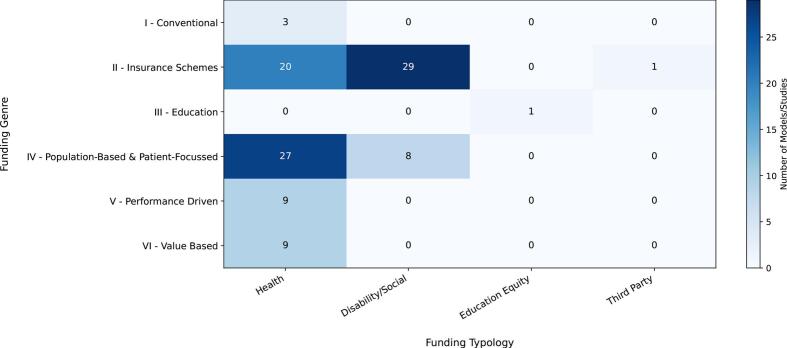
Heatmap of 86 included articles: Funding genre by funding typology (frequency). Note. The heatmap highlights 107 (n = 107) funding genres. This is higher than the 86 articles (n = 86) identified in the scoping review as each article may identify more than one funding genre.

Fig. 3 presents a heatmap from the identified evidence, highlighting the distribution of six funding genres across the four identified typologies. Notably, the majority of identified typologies were classified within the insurance scheme FM genre, with limited examples mapped to other genres. Darker blue shading reflects a higher frequency of studies or PFMs classified within each genre/typology pairing. The analysis indicates that insurance scheme (Genre II) and population-based and patient-focussed (Genre IV) approaches dominate the current evidence base. Collectively, these two categories represent the dominant genres identified across funding typologies within the extracted literature corpus (i.e., within the health and disability/social sectors). By contrast, conventional (Genre I) and education (Genre III) models were far less represented in the literature. Performance driven (Genre V) and value-based (Genre VI) models show modest representation within the health sector (with no examples for each genre in the disability/social sector) indicating an emerging focus on outcome-driven frameworks. Limited research was observed within the education equity and third-party typologies across all genres, highlighting ongoing limitations in the evidence base beyond health settings. In synthesising these findings, Fig. 3 demonstrates that the scientific evidence base for PFMs remains heavily weighted toward health-system reforms favouring insurance schemes (Genre II) and population-based and patient-focussed (Genre IV) approaches, with emerging uptake of performance-oriented (Genre V) and value-based (Genre VI) approaches.

### 2.8 Funding model genres

The appraisal of the literature identified the following categorisation of FMs according to the following genres (see Table 2): (1) *conventional*; (2) *insurance schemes*; (3) *education;* (4) *population-based and patient-focussed*; (5) *performance-driven;* and (6) *value-based.*

#### 2.8.1 Conventional funding models

Of the 86 studies identified, three studies (*n* = 3, 3.5 %) (studies 53, 61, 63) outlined four conventional FMs which are underpinned by varying degrees of funding mechanisms and provisions: (a) the Beveridge model; (b) the Bismarck model; (c) the National Health Insurance model; and (d) the Out-of-Pocket model. Two studies (*n* = 2, 2.3 %) (studies 61, 63) detailed how funding provisions are typically arranged according to conventional FMs. These studies identified several advantages and disadvantages of conventional funding models. Advantages included predictable revenue (e.g., fee-for-service), simple financial planning, established and widely understood frameworks, flexibility based on services delivered, and incentives for high-service volume. Disadvantages included limited emphasis on prevention, incentives for unnecessary treatment, prioritisation of quantity over quality, cost inefficiencies from episode-based rather than whole-system care, and inequities related to socioeconomic status and geographic access.

#### 2.8.2 Insurance schemes

The review of the literature identified one seminal article which described an array of insurance schemes used within primary care settings [8] (study 12). This study classified FMs according to five FM typologies (i.e., traditional disease, expansion, prevention/personal responsibility, and potential/age rationing), where SLP aligns to the traditional disease, enhancement and prevention typologies. Crigger [8] examined the justification of resource allocation, evaluating FMs based on their effects on quality of care, financial viability, and accessibility. The paper emphasises the professional obligation to balance funding and policy decisions to maximise equitable access and promote the common good [8]. Of significance to this paper is Busse et al. [10]’s framework outlining the health financing arrangements of twenty-five high-income countries (study 6). The paper presents the theoretical functions of health financing systems together with the various FM mechanisms and funding flows (see introduction). Busse et al. [10] anchors the analysis in a well-established theoretical architecture for understanding how financing arrangements drive system behaviour and reform.

Other insurance schemes identified in the literature search which are employed by primary care included *blended funding,* and a mix of *fee-for-service*, *out-of-pocket, capitated funding, pay for performance,* and *fixed payments per unit of time* (*n* = 8, 9.3 %) (studies 2, 3, 16, 48, 55, 56, 66, 79). Within an Australian context, the literature described a range of PFMs available for use within Australia’s independent SLP sector: Medicare’s Chronic Disease Management Plan initiative (*n* = 11, 12.8 %) (studies 1, 8, 9, 23, 24, 29, 33, 39, 57, 71, 72); Medicare’s Helping Children with Autism and Better Start for Children with Disability initiatives (*n* = 5, 5.8 %) (studies 14, 19, 20, 45, 82); NDIS (*n* = 9, 10.4 %) (studies 14, 19, 25, 30, 34, 45, 64, 65, 82); veteran affairs (*n* = 1, 1.2 %) (study 47); and mental health initiatives (*n* = 1, 1.2 %) (study 66). Twenty studies discussed flexible funding mechanisms (*n* = 20, 23.3 %), incorporating direct funding provisions (*n* = 11, 12.8 %) (studies 5, 26, 28, 30, 31, 44, 58, 67, 75, 76, 84), self-determined funding arrangements (*n* = 6, 7.0 %) (studies 5, 20, 35, 49, 67, 70), and individualised FMs (*n* = 12, 14.0 %) (studies 21, 26, 34, 40, 41, 42, 44, 64, 67, 74, 75, 84). Currently, Australian families can access independent SLP services through the above-mentioned PFMs.

#### 2.8.3 Education equity adjustment funding models

The literature search identified a single article by Vlcek et al. [132] (study 81), which examined collaborative partnerships between teachers, parents, and allied-health professionals within inclusive education contexts. The article discussed education funding mechanisms designed to support students with autism spectrum disorder and highlighted the central role of cross-sector collaboration in promoting participation and learning. Notably, the authors reported that constrained funding significantly affects mainstream teachers’ capacity to respond to the diverse and individualised needs of students with ASD. Educational equity requires targeted funding arrangements (such as needs-based loadings and inclusive education supports) to resource appropriate adjustments and reduce structural barriers within mainstream settings.

#### 2.8.4 Population-based and patient-focussed funding models

FMs that consider characteristics of a population (i.e., socioeconomics including geographic location, population size, and community healthcare needs) can be arranged according to two main funding typologies: (a) *population-based*; and (b) *patient-focussed.* Of the 86 studies identified, 17 studies (*n* = 17*,* 19.8 %) detailed how services were funded and delivered according to the needs at a population level (studies 4, 10, 11, 16, 17, 18, 22, 38, 43, 50, 51, 52, 54, 56, 60, 73, 86). Population-based funding allocates block payments to providers based on the size and needs of a defined population, considering factors such as age, socioeconomic status, ethnicity, sex, and rural or regional location. It is used across primary care, hospitals, community care, mental health, and aged care (studies 51 & 56). Advantages include support for prevention and early intervention, greater equity, efficient resource use, flexible service design, and integrated care. Disadvantages include risks of underfunding if needs are not accurately assessed, inequitable distribution, implementation complexity, reduced care quality if costs are prioritised, and limited responsiveness to changing population needs.

Two studies examined population-based funding from a multinational perspective (n = 2, 2.3 %). A comparative analysis of seven international tertiary healthcare models (study 60) found strong alignment in population-need factors and cost adjustments for market influences such as public/private mix, rurality, teaching and research. A systematic review across 15 countries (study 11) found some jurisdictions adjusted funding formulae for disadvantage and ethnicity, but none accounted for cultural and linguistic diversity or health literacy. The authors recommended governments adopt broader, more equitable population-need criteria when reviewing FMs. Multiple studies described block funding (*n* = 14, 16.3 %) (studies 1, 8, 9, 14, 23, 24, 29, 33, 39, 57, 71, 72, 81, 82), a system that provides fixed amounts based on historical and population-based funding measures. Many core SLP services within the health, education, and disability sectors receive funding via block funding methods (e.g., Independent Schools Victoria [141], the former Early Intervention – Helping Children with Autism, and Better Start initiatives where blocks of funding up to AU$12,000 per child were provided [142]).

Fourteen studies (*n* = 14, 16.3 %) outlined how *patient-focussed FMs* are used in instances where service providers use incentives and supports such as financial incentives, further education, and training to improve the quality and efficacy of care for individuals with a range of health needs (studies 1, 8, 9, 15, 16, 23, 24, 29, 33, 39, 57, 59, 71, 72). According to Oliver-Baxter and Brown [48], patient-focussed FMs tailor treatment to individual needs, align care with patient goals, support early intervention, improve efficiency through targeted and coordinated care, and prioritise patient-centred outcomes over service volume (study 56). Reported disadvantages included complexity in design and implementation, potential inequity for vulnerable groups, high implementation costs, greater administrative demands, and tension between cost control and quality care (study 56). Oliver-Baxter & Brown [48] described examples of patient-focused FMs in Australia, which included *fee-for-service models* where providers charge for individual service items they provide, *pay-for-performance models* where payments to professionals or practices are derived from the type or number of services provided (e.g., Medicare’s Chronic Disease Management Plan [studies 1, 8, 9, 23, 24, 29, 33, 39, 57, 71, 72]); and *activity-based funding* where providers are subsidised based on expected activity (i.e., expected costs for clinically defined episodes of care; see also *performance-driven FMs* [studies 15, 16, 59]).

Five studies which incorporated a mix of population-based and patient-focused FMs illuminated the challenges facing individuals and families when accessing services in rural and remote areas in Australia (*n* = 5, 5.8 %) (studies 14, 26, 34, 36, 76), with one study (*n* = 1, 1.2 %) (study 76) also describing the barriers faced by Aboriginal and Torres Strait Islander people in accessing funding provisions. As reported in these studies, the challenges facing individuals in accessing timely and appropriate healthcare in rural, regional, and remote areas included the availability and affordability of care, the difficulties in accessing services due to provider shortages and their skill level(s), the training and capacity of carers, and the inability to engage with other individuals with like healthcare needs due to the relative isolation and tyranny of distance in living regionally or remotely.

#### 2.8.5 Performance-driven funding models

To provide a comprehensive overview of FM genres, the inclusion criteria exposed a range of performance-driven funding formulae which applied to a range of settings relevant to SLP service delivery. Global health-systems (including Australian allied-health sectors) have been historically funded via three main funding streams: activity-based/casemix; block; and other funding (i.e., cross-border flows, interest, public-health funding streams). The literature search identified eight studies (n = 8, 9.3 %) (studies 7, 15, 16, 46, 59, 69, 78, 80) that described performance-driven funding arrangements (i.e., activity-based/casemix funding mechanisms) relevant to allied-health settings. The eight studies outlined a range of benefits and limitations generated through the implementation of performance-driven FMs. As outlined by Duckett [76] and Palmer et al. [35], the underlying premise of activity-based funding was the promotion of efficiencies and transparency by merging funding provisions with increased service output, whereby providers were encouraged to meld efficiencies in resource allocation with innovative service design and delivery (studies 16 and 59). Palmer et al. [35] reported that limitations of activity-based funding included incentives for over-servicing, unnecessary administrative complexity, and unintended inequities in access for vulnerable populations affected by geographic and socioeconomic disadvantage (study 59). Conversely, Byron & McCathie [69] reported that casemix funding accommodated adjustments in funding provisions based on patient complexity and healthcare needs (study 7). According to Byron & McCathie [69], casemix funding aimed to align patient needs with equitable resource allocation; however, Turner-Stokes et al. [51] asserted limitations due to its potentially high administrative burden and funding disparities between populations with differing complexities (study 80).

#### 2.8.6 Value-based funding models

Over the past two decades*, value-based funding* arrangements have received traction in the literature and captured the attention of international healthcare policy architects. Whilst the literature extensively reported *value-based healthcare*, distinguishing it from evidence on value-based funding mechanisms and arrangements was paramount. The literature search, therefore, identified nine pieces of evidence (*n* = 9, 10.5 %) exploring value-based funding arrangements (studies 13, 27, 32, 37, 62, 68, 77, 83, 85). According to Wise et al. [136], this FM genre sought to enhance the outcomes considered essential by patients, by reducing reliance on low-value interventions and modifying services and models of care to optimise integration between providers and patient-directed improvements (study 85). According to Wise et al. [136], an aspiration of value-based funding has been to implement change of funding arrangements within existing payment structures to facilitate efficiencies (study 85). Within an Australian context, several white papers have proposed FM policy reform through the application of value-based funding arrangements across settings (studies 13, 32, 62). An advantage of value-based funding is that resources are allocated according to patient value and outcomes rather than service volume (studies 27, 37). A core design feature is the transfer of financial risk from funders and payers to providers, who are responsible for reducing costs while improving patient outcomes (studies 13, 37, 85).

Supplementary Material III (Summary of evidence – Australian public funding models for paediatric access pathways) provides a synthesis of the reviewed evidence, mapping the classified funding genres and typologies directly to the specific access pathways through which Australian children, young persons and their families receive SLP services.

### 2.9 Discussion

For individuals and families accessing SLP services, funding is a critical consideration and often determines continuity of healthcare access [2], [88], [143]. Given the soaring cost of healthcare services in Australia [144], individuals with communication and swallowing difficulties and their families may refrain from accessing critical services based on the availability (or absence) of full or partial public funding [145], [146]. Furthermore, the variability of cost to access services by individuals and families [20], [21], [22], [147] may prevent some from accessing care. Families sensitive to high healthcare expenses, therefore, require support from the public purse via public funding provisions to access timely and critical care. Public funding, as defined by the results from this literature review and the identification of six PFM genres, therefore provides critical support to individuals and families in need of SLP services.

A significant contribution of this research was the clarification of a *healthcare FM* [2]. The lack of clarity in the initial empty review, combined with the high degree of variation observed across studies in the scoping review, necessitated a systematic interpretation of the term *healthcare funding* (see Supplementary Material IV − Clarification of healthcare funding model). This process led to the classification of PFMs and revealed the interlocking concepts of *funding genres* and *typologies*. Despite an extensive and comprehensive search of the literature, a precise and universally accepted definition of a healthcare FM was not identified. Supplementary Material IV (Clarification of healthcare funding model) presents our proposed clarification [2], synthesising the foundational funding principles articulated by Busse et al. [10], Kurtzin [148], Murray & Frenk [6], and the World Health Organization [7]. A healthcare FM is conceptualised as encompassing the range of mechanisms and provisions (such as flexible, self-determined, or individualised funding) through which money allocated for healthcare (i.e., pooled funds) is reserved for the final transaction to remunerate service delivery (see Supplementary Material IV – Clarification of healthcare funding model). Distinguishing between the broader construct of *health financing* and the more specific domain of *healthcare funding* has refined the scope of this research, enabling targeted investigation of FMs pertinent to SLP. Moreover, this conceptual clarification strengthens future research by delineating the micro-elements of a FM, including its processes, mechanisms, provisions, funding flows, and the ultimate destination of resources.

This review distinguished between (a*) funding model genre*, and (b) *funding model typology*. As summarised in Table 1, *funding model genre* refers to the classification of PFMs according to defined characteristics such as the structure, architecture and aspiration of the model (e.g., population-based versus performance-driven PFMs). Conversely, *funding model typology* refers to the systematic classification of PFMs as a consequence of their distinct mechanisms and overall function and purpose (e.g., health versus social insurance arrangements). An important finding from this scoping review is the identification that PFMs can be categorised according to FM genres and/or FM typologies. Table 1 presents the profiling and categorisation of six PFM genres together with a range of PFM typologies relevant to SLP. The prominence of both insurance schemes (Genre II) and population-based and patient- focussed (Genre IV) approaches reflects broader governmental and policy movements toward multidisciplinary, coordinated care models within health and social systems (Fig. 3). This concentration reflects recent government policy shift towards funding mechanisms that prioritise accessibility, flexibility, and responsiveness to client needs. Conversely, the relative scarcity of conventional (Genre I) and performance-driven (Genre V) models suggests that these approaches are either being discontinued/phased out due to their declining policy relevance or remain under-investigated. The moderate but expanding representation of value-based (Genre VI) models, points to an evolving but important trend toward linking funding with measurable outcomes and quality indicators. The limited evidence from education equity and third-party typologies highlights a persistent sectoral imbalance in PFM research; the near-absence of research in these areas highlights the need for greater exploration of how educational and compensatory PFMs align with insurance schemes and value-based funding. This gap constrains the ability to generalise findings across diverse service contexts and limits understanding of how funding design influences cross-sector collaboration. Future research should extend beyond the health sector to achieve a more comprehensive understanding of funding diversity and the impact of PFM mechanisms on service provision (i.e., accessibility, equity, efficiency and acceptability; [2]).

### 2.10 The fluidity of public funding models

The literature review also identified that PFMs are fluid (refer to Fig. 3); that is, PFMs can be categorised across FM genres and/or typologies due to their structure, mechanisms, and aspirational outcomes (e.g., Medicare’s Chronic Disease Management Plan is a health insurance model but can also be categorised as a patient-focused PFM [see studies 1, 8, 9, 23, 24, 29, 33, 39, 57, 71, 72]), and activity-based funding can be categorised as both a patient-focused PFM and a performance-driven PFM (see studies 15, 16, 59). Twenty-three out of the 86 studies (*n* = 23, 26.7 %) integrated two FM classifications (i.e., *FM genre and/or typology*): (a) insurance type FMs and population-based and patient-focussed FMs (studies 1, 8, 9, 14, 19, 23, 24, 26, 29, 33, 34, 35, 39, 56, 57, 58, 71, 72, 76, 82); and (b) patient-focussed FMs and performance-driven FMs (studies 15, 14, 59). In addition, there was one study by an international expert on performance-driven FMs ([76]; *n* = 1, 1.2 %) (study 16) that analysed three FM genres relevant to allied-health (i.e., insurance schemes, population-based and patient-focussed, and performance-driven FMs) across two countries, Australia and Canada. This evidence suggests that PFM policy architects are able to integrate mechanisms from a range of funding genres to design population-specific public funding arrangements. However, these PFMs are not without limitations given the range of constraints (e.g., limited funding versus the required number of sessions to achieve outcomes, potential for inequity due to socioeconomic circumstances [i.e., geographic location], potential for out-of-pocket gap payments after PFM rebates, risks of some PFMs where quantity over quality of care is financially rewarded).

### 2.11 Diversity of Australian funding formulae

**Fig. 4:**
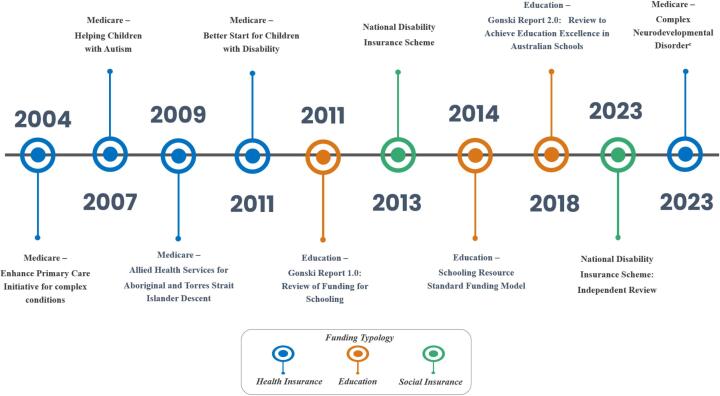
A selection of Australian public funding initiatives: The evolution of access pathways to paediatric speech-language pathology services^a^ Note. ^a^The following public funding initiatives were added to the Medicare Benefit Schedule (MBS): (a) Enhanced Primary Care Plan initiative (name changed to Chronic Disease Management Plan) was introduced in 2004; (b) in 2007, Helping Children with Autism was added to the MBS; (c) in 2011, Better Start for Children with Disabilities was added to the MBS; (d) in 2023, Complex Neurodevelopmental Disorder & Eligible Disabilities was added to the MBS and superseded both Helping Children with Autism and Better Start for Children with Disabilities [150]. In 2026, speech sound disorders, stuttering and cleft palate SLP services will be added to this MBS item. Also refer to Nickless [2, Table 8.2] for corresponding policy, legislation and major reviews. Thriving Kids is a proposed national PFM led by the Australian Federal Government, with staged rollout of state-based services commencing by October 2026 and full implementation projected for January 2028. At the time of writing this article, the final structure of this PFM is yet to be defined.

Australia is dependent on a mixed system of funding allocations for health, education, disability and community sectors which incorporate elements from both welfare and market models [140], [149]; that is, the healthcare system is a mixed model of public and private health services, with the overarching architecture based on principles of universal access. This scoping review identified a broad range of PFMs used to offset out-of-pocket cost barriers and to facilitate access to independent SLP services in Australia. Australia’s public funding landscape is highly varied, with multiple models introduced over time (Fig. 4) in response to shifting policy priorities, resulting in diverse access experiences for children, young persons and their families. Further changes and additions are anticipated in 2026, including the introduction of Thriving Kids.

In Australia, public funding is allocated by governments and other organisations for the purpose of individuals with communication and swallowing difficulties accessing timely services from the independent sector [2], [140], [151], [152]. This scoping review has identified six PFM genres (i.e., conventional, insurance schemes, education, population-based and patient-focussed, performance-driven and value-based funding) and several related typologies (i.e., health/social/third-party insurance; and education funding). In analysing the literature, the results illuminated how PFMs offer a range of advantages for children, young persons, and their families. For example, insurance scheme funding arrangements offer flexible and individualised funding arrangements (e.g., NDIS), and patient-focused PFMs tailor treatments to an individual’s healthcare needs and encourage early intervention which supports preventative healthcare (e.g., Chronic Disease Management Plan). However, PFMs also present several disadvantages. Examples include incentives for providers to perform more treatments and services than necessary rather than improving quality of care (e.g., NDIS); funding being inadequate at an individual level to resource intervention scale required to achieve outcomes (e.g., Independent Schools Victoria; Chronic Disease Management Plan), and inequity when access is challenging for vulnerable populations (i.e., due to socioeconomic circumstances, including geographic location). These considerations are important to explore in the context of the allocation of funding, particularly when predicting the advantages of PFMs, and identifying how to safeguard against disadvantages (i.e., safeguarding public funding to non-government entities and how to ensure increasing emphasis on performance evaluation and deliverables such as access, efficiency, acceptability, and performance evaluations and outcomes rather than inputs) [153], [154]. Evaluation of contemporary PFMs against health-economic objectives of equity, efficiency, and acceptability [2] is a critical step in addressing these safeguard questions.

### 2.12 The impact of Australian PFMs for speech-language pathology

Since 2004, successive Australian PFMs, particularly through the Medicare Benefits Schedule (MBS), have expanded subsidised access to SLP services (Fig. 4). These reforms signal growing policy recognition of communication and swallowing disorders; however, their implementation highlights persistent challenges in translating funding intent into equitable service delivery. Structural features such as capped service entitlements (e.g., five sessions per annum under MBS item 10,970 [see Supplementary Material V – Medicare Benefit Schedule, speech-language pathology items]; Independent Schools of Victoria’s allocation of $800 per student for core SLP services), general practitioner gatekeeping, and variability in referral practices constrain access and limit the effectiveness of patient-focussed purchasing arrangements [2]. Evidence indicates that session limits are misaligned with recommended treatment dosage and intensity, raising concerns regarding policy–practice incongruence and socio-demographic inequities [2]. Collectively, these findings point to a fragmented funding architecture and underscore the need for stronger system stewardship and cross-sector integration to align pooling, purchasing, and provision functions with the scientific evidence base required to optimise SLP outcomes [2].

### 2.13 Why is it important to understand healthcare funding models?

Understanding the aetiology of SLP funding is important for a range of stakeholders involved in the delivery of SLP services. In an Australian context, the profession of SLP is currently self-regulated, whereby members of the peak body representing speech pathologists, Speech Pathology Australia (SPA), regulate the clinical, professional and ethical standards of members [155]. Practising membership of SPA is a requirement for a range of funding provisions, including access to the MBS as a Medicare provider and the NDIS [155]. Moreover, inclusion of pre-service training for entry-level speech-language pathologists is essential, as many paediatric (and adult) SLP services involve public funding provisions; Speech Pathology Australia’s Code of Ethics [156] requires members to “act with integrity, diligence, and honesty when accessing and managing funding provisions for our services.” [156, s. 2.4 Service planning and provision]. An imperative exists for speech-language pathologists to have knowledge of current funding systems and sources in order to optimise their response to clients' needs. This is an ethical obligation [156].

### 2.14 Future research: Knowledge of Australian funding outcomes

Little is known regarding which FMs are congruent with the best scientific evidence available for SLP management [2]. As is the case internationally and in Australia, the delivery of SLP services is limited by the financial resources made available by government and other funders (i.e., private health insurers, third parties such as charities). Although the SLP profession has emerging evidence to support the value of intervention in a number of areas of clinical practice, there exists limited information surrounding the cost of providing these services, and which FMs and sources of funding (e.g., government funding, private funding or other) are most effective.

All tiers of government are challenged to fund and, in some cases, provide SLP services in a timely and appropriate setting [140]. The limitations of funding for service provision impact on the social, health, education, and economic outcomes of individuals with communication and language difficulties [157], [158], [159], [160]. Communication and swallowing disorders impose significant costs (not only financial, but also health and social costs) to an individual and, subsequently, to the greater community. Preliminary analysis of SLP costs within an Australian context has been documented in the literature [20], [21], [22], [25], [161]. In addition to an improved understanding of cost of SLP services, further investigation into the models of funding, their alignment with principles of Differential Treatment Intensity [2], [162], [163] and those providing optimal outcomes is required.

A recently proposed national PFM, Thriving Kids, is under development by the Australian Federal Government, with staged rollout of state-based services scheduled to commence by October 2026 and full implementation anticipated by January 2028. At the time of writing this paper, the FM’s final structure and operational arrangements remain to be determined; nonetheless, it is essential that the PFM’s design explicitly incorporates health economic objectives of equity, efficiency, and acceptability [2], [140].

### 2.15 Conclusion

Within an Australian context, SLP services are embedded within a multifarious network of health, education, disability, and community settings. Within each of these sectors, funding of SLP services is complex and does not come from one single source. Current public funding arrangements via independent practice of core SLP services are delivered via a range of *FM genres* and *typologies*. The use of public funding allocations in accessing independent SLP services in Australia by children, young persons, and their families for communication and swallowing management services is enviable when compared internationally. However, there is a continued need to investigate how current funding provisions across all settings align with evidence-based intervention and management guidelines to meet desired outcomes for children and young persons with communication and swallowing difficulties.

## 3 Declaration of generative AI and AI-assisted technologies in the manuscript preparation process

During the preparation of this work the author(s) used ChatGPT in order to generate the heatmap in Figure III from data collected during the research process. After using this tool/service, the author(s) reviewed and edited the content as needed and take(s) full responsibility for the content of the published article.

## CRediT authorship contribution statement

**T. Nickless:** Writing – original draft, Validation, Software, Methodology, Investigation, Formal analysis, Conceptualization. **B. Davidson:** Writing – review & editing, Supervision. **L. Gold:** Writing – review & editing, Supervision. **R. Dowell:** Writing – review & editing, Supervision, Conceptualization.

## Declaration of competing interest

The authors declare that they have no known competing financial interests or personal relationships that could have appeared to influence the work reported in this paper.
