# Supplementary material for: Genres and typologies of standard paediatric service public funding model provisions for speech-language pathology management: A scoping review
Source: Health Policy Open. 2026 May 19;11:100173. doi: 10.1016/j.hpopen.2026.100173 (PMC13260217; doi:10.1016/j.hpopen.2026.100173)
Supplement: Supplementary Data 1 [file mmc1.docx]

# ***Supplementary Material I***

# ***Literature search strategy***

The literature search included the following line of inquiry using Boolean and MeSH (medical subject headings) search retrieval terms. These queries included:

- word variations (i.e., paediatric, children, adolescent; National Disability Insurance Scheme, NDIS, Disability Insurance)
- truncations (i.e., therap* for therapy, therapies, therapist, therapists),
- use of word proximity (e.g., fund* adj3 children [for OVID] and fund* N3 children [for CINAHL]); and
- wildcards (e.g.,? and $ for various spellings of words containing ‘s or z’ such as organi$ation).

The following search strategy provides a sample list of terms used in the scoping review (not an exhaustive list):

- Fund* AND healthcare NOT research; health service* fund*; self directed fund* AND healthcare; fund* health system*; fund* model AND health NOT research
- Enhanced primary care; Chronic Disease Management Plan; National Disability Insurance Scheme fund*; Medicare; public fund* AND privati?sation; fund*; Gonski AND disability
- Individuali$sed fund*; population based fund*; blended fund* AND healthcare; capitation fund* AND healthcare; funding mechanism* AND healthcare; social insurance AND Australia

Table A.1 and A.2 provide a sample of the search strategy is detailed below:

***Table A.1: Sample database search for PubMed***

| 57 | "gonski" and "disability" | | |
| --- | --- | --- | --- |
| 53 | #52 and #2 | |  |
| 52 | NDIS |  |  |
| 51 | #50 and #2 | |  |
| 50 | National disability insurance scheme | | |
| 49 | #47 an #2 |  |  |
| 48 | #47 and #11 | |  |
| 47 | "chronic disease management initiative" and "australia" | | |
| 46 | "chronic disease management plan" and "australia" | | |
| 45 | #44 and # 2 | |  |
| 44 | "chronic disease management" and "australia" | | |
| 43 | #42 and #2 | |  |
| 42 | "enhanced primary care" and "australia" | | |
| 41 | #40 and #2 | |  |
| 40 | "medicare" and "australia" | | |
| 39 | #38 and #2 | |  |
| 38 | "person centred care" and "funding" | | |
| 37 | "public fund*" and "privatization" | | |
| 36 | "public health* adj2 funding" | | |
| 35 | "public healthcare funding" and "privati?ation" | | |
| 34 | "public healthcare funding" and "privati$ation: | | |
| 33 | "social insurance" and "healthcare" | | |
| 31 | "individuali?ed funding" | | |
| 30 | "individuali$ed funding" and "healthcare" | | |
| 29 | "population based funding" | | |
| 28 | "Funding model" and "healthcare" | | |
| 27 | #26 AND #2 | |  |
| 26 | #25 AND #19 | |  |
| 25 | Fund* system* | |  |
| 24 | #23 AND #2 | |  |
| 23 | #22 not "research" | |  |
| 22 | #21 and #19 | |  |
| 21 | fund* mechanism* | | |
| 20 | #18 & #19 | |  |
| 19 | healthcare | |  |
| 18 | self directed funding | | |
| 17 | #16 and #11 | |  |
| 16 | #15 and #13 | |  |
| 15 | public health funding | | |
| 13 | health services funding | | |
| 12 | #8 and #11 | |  |
| 11 | allied health | |  |
| 8 | #7 and #2 |  |  |
| 7 | #1 not #3 |  |  |
| 3 | low middle income | |  |
| 2 | children |  |  |
| 1 | (funding) AND (healthcare) | | |

***Table A.2: Sample database search for CINAHL***

| "chronic disease management plan"[All Fields] AND "australia"[All Fields] |
| --- |
| ("chronic disease management"[All Fields] AND "australia"[All Fields]) AND "2"[All Fields] |
| "chronic disease management"[All Fields] AND "australia"[All Fields] |
| "enhanced primary care"[All Fields] AND "australia"[All Fields] AND ("child"[MeSH Terms] OR "child"[All Fields] OR "children"[All Fields] OR "child s"[All Fields] OR "children s"[All Fields] OR "childrens"[All Fields] OR "childs"[All Fields]) |
| "enhanced primary care"[All Fields] AND "australia"[All Fields] |
| "medicare"[All Fields] AND "australia"[All Fields] AND ("child"[MeSH Terms] OR "child"[All Fields] OR "children"[All Fields] OR "child s"[All Fields] OR "children s"[All Fields] OR "childrens"[All Fields] OR "childs"[All Fields]) |
| "medicare"[All Fields] AND "australia"[All Fields] |
| "person centred care"[All Fields] AND "funding"[All Fields] AND ("child"[MeSH Terms] OR "child"[All Fields] OR "children"[All Fields] OR "child s"[All Fields] OR "children s"[All Fields] OR "childrens"[All Fields] OR "childs"[All Fields]) |
| "person centred care"[All Fields] AND "funding"[All Fields] |
| "public funding"[All Fields] AND "privatization"[All Fields] |
| "public healthcare funding"[All Fields] AND "privatization"[All Fields] |
| "public healthcare funding"[All Fields] AND "privatisation"[All Fields] |
| "social insurance"[All Fields] AND "healthcare"[All Fields] |
| individuali$ed funding |
| individuali$ed funding and healthcare |
| "population based funding"[All Fields] |
| "Funding model"[All Fields] AND "healthcare"[All Fields] |
| ("economics"[MeSH Subheading] OR "economics"[All Fields] OR "funding"[All Fields] OR "economics"[MeSH Terms] OR "financial management"[MeSH Terms] OR ("financial"[All Fields] AND "management"[All Fields]) OR "financial management"[All Fields] OR "funded"[All Fields] OR "funds"[All Fields] OR "fund s"[All Fields] OR "fundings"[All Fields]) AND ("drug delivery systems"[MeSH Terms] OR ("drug"[All Fields] AND "delivery"[All Fields] AND "systems"[All Fields]) OR "drug delivery systems"[All Fields] OR "system"[All Fields] OR "system s"[All Fields] OR "systems"[All Fields]) AND ("delivery of health care"[MeSH Terms] OR ("delivery"[All Fields] AND "health"[All Fields] AND "care"[All Fields]) OR "delivery of health care"[All Fields] OR "healthcare"[All Fields] OR "healthcare s"[All Fields] OR "healthcares"[All Fields]) AND ("child"[MeSH Terms] OR "child"[All Fields] OR "children"[All Fields] OR "child s"[All Fields] OR "children s"[All Fields] OR "childrens"[All Fields] OR "childs"[All Fields]) |
