# Supplementary material for: Genres and typologies of standard paediatric service public funding model provisions for speech-language pathology management: A scoping review
Source: Health Policy Open. 2026 May 19;11:100173. doi: 10.1016/j.hpopen.2026.100173 (PMC13260217; doi:10.1016/j.hpopen.2026.100173)
Supplement: Supplementary Data 2 [file mmc2.docx]

Supplementary Material II

Characteristics of evidence (86 included studies)

| **Characteristic** | ***n (Total n =86)*** | **Percent** |
| --- | --- | --- |
| **Geographic region (including multinational)**  Australia  Multinational  New Zealand  United Kingdom  United States/ United States & Canada | 57  12  4  5  8 | 66.3  14.0  4.7  5.8  9.3 |
| **Funding model typology^a^**  Health  Disability/ Social  Education  Third Party | 53  31  1  1 | 61.6  36.0  1.2  1.2 |
| **Levels of evidence^b^**  Level 1 Qualitative or mixed methods systematic reviews  Level 2 Qualitative or mixed methods synthesis  Level 3 Single qualitative study  Level 4 Systematic review of expert opinion  Level 5 Expert Opinion | 4  17  28  1  36 | 4.6  19.8  32.6  1.2  41.9 |

*Note. For the purpose of decimal rounding, cumulative percentages may not equal 100; ^a^adapted from Crigger [8]. Also refer to Table I in [140]; ^b^Levels of Evidence for Meaningfulness [138,139]. Refer to main article for references.*
