# Supplementary material for: Genres and typologies of standard paediatric service public funding model provisions for speech-language pathology management: A scoping review
Source: Health Policy Open. 2026 May 19;11:100173. doi: 10.1016/j.hpopen.2026.100173 (PMC13260217; doi:10.1016/j.hpopen.2026.100173)
Supplement: Supplementary Data 3 [file mmc3.docx]

Supplementary Material III

Summary of evidence - Australian public funding models for paediatric access pathways

|  | **Funding typology** | | | |
| --- | --- | --- | --- | --- |
| **Funding genre** | **Health** | **Disability/Social** | **Education**  **Equity** | **Third Party** |
| **Conventional** | — | — | — | — |
| **Insurance schemes** | Medicare:   - CDMP^a^ - HCWA^b^ - BS^c^ - ATSI^d^ | NDIS^g^ | — | Third Party^i^ |
| **Education** | — | — | Independent School^h^ | — |
| **Population-based & patient-focussed** | Medicare:   - CDMP^a^ - HCWA^b^ - BS^c^ - ATSID^d^ | — | Independent School^h^ | — |
| **Performance-driven** | Activity based/ casemix^e^ | — | — | — |
| **Value-based** | Hospital value-based care^f^ | — | — | — |

*Notes. Due to the fluid nature of Australian PFMs, some PFMs fall into different funding genres. Refer to Table 8.2 in Nickless [2] for an overview including major review(s) and associated outcomes of PFM policy and legislation for each corresponding funding typology. Also refer to Nickless et al. [152, Supplementary File III] for PFM criteria and explanations. ^a^CDMP = Medicare - Chronic Disease Management Plan; ^b^HCWA = Medicare -Helping Children with Autism; ^c^BS = Medicare - Better Start for Children with Disability; ^d^ATSID = Medicare – Allied Health Services for Aboriginal and Torres Strait Islander Decent; ^e^Funding within hospital and some community health settings; ^f^Three whitepapers identified in the review proposed value-based funding models in healthcare settings [52,91,115]; ^g^NDIS = National Disability Insurance Scheme; ^h^State based funding mechanisms used for Independent Schools Victoria; ^i^Third Party = Department of Veteran Affairs, workers’ compensations insurance schemes and transport accident commissions. Refer to main article for references.*
