# Supplementary material for: Genres and typologies of standard paediatric service public funding model provisions for speech-language pathology management: A scoping review
Source: Health Policy Open. 2026 May 19;11:100173. doi: 10.1016/j.hpopen.2026.100173 (PMC13260217; doi:10.1016/j.hpopen.2026.100173)
Supplement: Supplementary Data 4 [file mmc4.docx]

Supplementary Material IV

***Clarification of healthcare funding model^a^***

**Healthcare Funding Model**

**… the *mechanisms* and *provisions* (e.g., from fund pooling to purchasing) by which *funds arrive* at a service or organisation as the *end-point* of the *health financing process***

*Note. ^a^[2]. Funding is like filling a piggy bank. You carefully deposit coins regularly, slowly accumulating wealth. Each contribution represents an investment in future possibilities. Similarly, funding involves consistent allocation of resources, nurturing growth, and patiently building a reserve to support endeavours when needed, ensuring an investment in the future. Refer to main article for references.*
