# Supplementary material for: Genres and typologies of standard paediatric service public funding model provisions for speech-language pathology management: A scoping review
Source: Health Policy Open. 2026 May 19;11:100173. doi: 10.1016/j.hpopen.2026.100173 (PMC13260217; doi:10.1016/j.hpopen.2026.100173)
Supplement: Supplementary Data 5 [file mmc5.docx]

Supplementary Material V

Medicare Benefit Schedule, speech-language pathology items

SLP services require more than the legislated allocation of five services per calendar year; the literature discusses dosage, frequency, and intensity of optimal SLP treatment outcomes beyond five services per calendar year [2]. This number of rebatable services under a combined health insurance and patient-focused PFM, such as the Chronic Disease Management Plan, imposes limitations on the intended structures and mechanisms of the PFM, thereby limiting optimal outcomes of SLP. It is important to consider that MBS subsidised services may still be biased by socio-demographic groups; for example, Figure 1 Supplementary Material V (Total number of services by age and gender: Medicare’s Chronic Disease Management Plan) highlights that MBS item 10970 is used for young children (<14 years) and more for boys than girls which may not fully match SLP population needs. Therefore, under such patient-focused arrangements, General Practitioners’ understanding of the scope of SLP practice beyond 14 years of age is narrow, resulting in limitation of service for other chronic and complex conditions relating to SLP. Funding of core SLP services under a restricted patient-focused model presents a range of limitations that counter the recommended dosage and intensity of intervention discussed in the literature. This review was unable to identify any studies aligning scientific evidence with PFM criteria.

Beyond the health sector, many SLP services within the education sectors are funded via block funding formulae. Independent Schools of Victoria’s funding for core SLP services (i.e., language, fluency, speech disorders) via an $800 per student funding allocation is an example of block funding [141]. Furthermore, the nature of the NDIS’s annual funding review process of individual support packages, although based on an insurance model, has the hallmarks of patient-focused formulae (e.g., tailoring intervention to an individual’s healthcare needs, facilitating early intervention and preventative measures, and advancing efficiencies through targeted interventions). Individualised funding packages are determined by National Disability Insurance Agency planners, who act as funding gatekeepers and allocate funding according to a pre-determined set amount of intervention, period of time (e.g., per annum), or specific program (e.g., Hanen programs or social skill intervention) in conjunction with healthcare providers. It is not within the scope of this paper to explain SLP services in detail; rather, these abovementioned services are provided as examples of core SLP services used within the constraints and complexities of Australia’s funding system.

Figure 1 Supplementary Material V

Total number of services by age and gender: Medicare’s Chronic Disease Management Plan


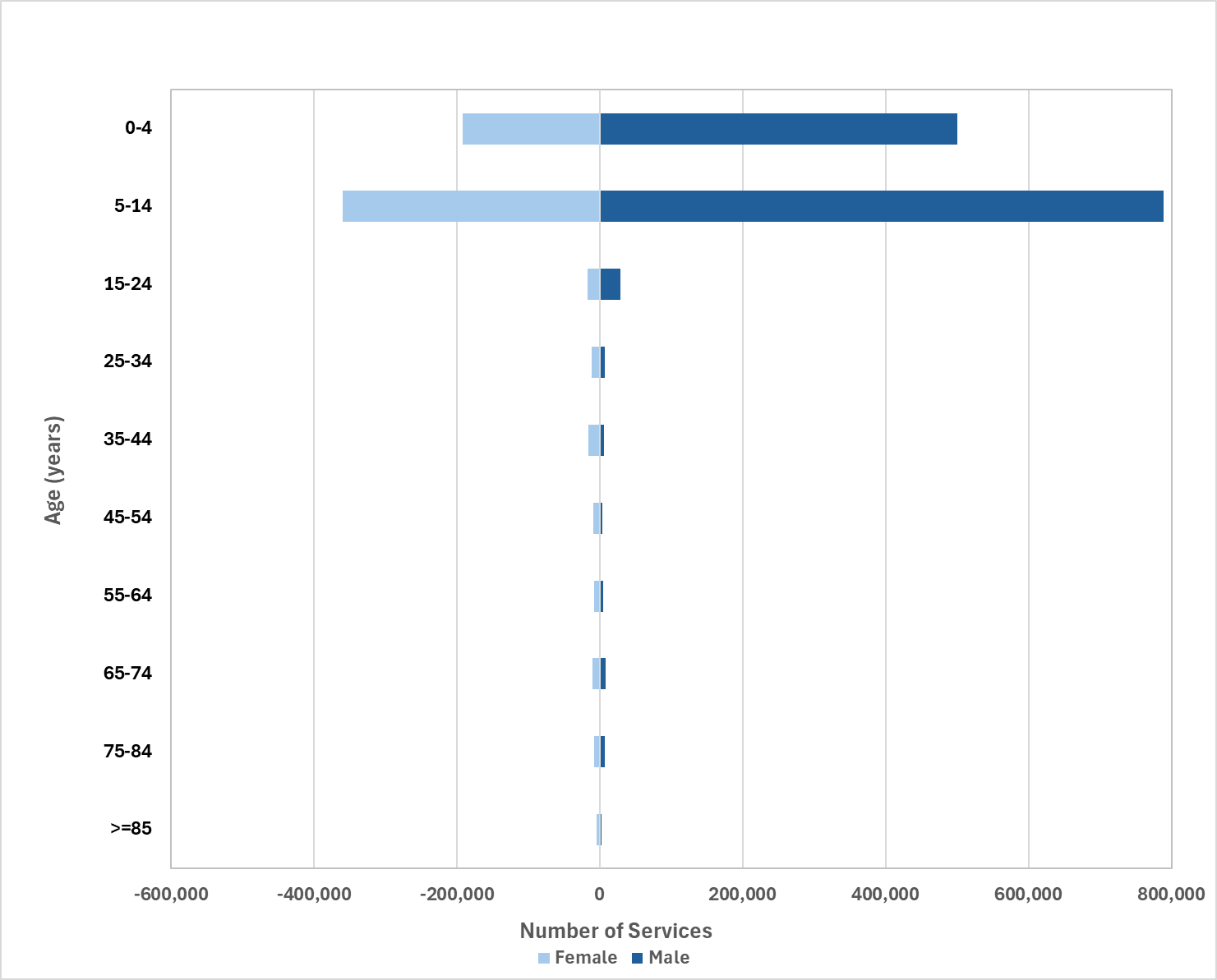


*Note. Total number of services by age and gender for Medicare Benefits Schedule item 10970 (speech-language pathology) under the Chronic Disease Management Plan (MBS CDMP), July 2004 to June 2024. Adapted from Services Australia, Medicare Item Reports [13]. Available from:* [*http://medicarestatistics.humanservices.gov.au/statistics/do.jsp?_PROGRAM=%2Fstatistics%2Fmbs_item_age_gender_report&group=10970&VAR=services&STAT=count&RPT_FMT=by+state&PTYPE=finyear&START_DT=200407&END_DT=202406*](http://medicarestatistics.humanservices.gov.au/statistics/do.jsp?_PROGRAM=%2Fstatistics%2Fmbs_item_age_gender_report&group=10970&VAR=services&STAT=count&RPT_FMT=by+state&PTYPE=finyear&START_DT=200407&END_DT=202406) *Copyright © 2024 Services Australia. Adapted with permission. References are provided in the main article.*

The introduction in 2004 of government-legislated rebates of core SLP services through the Medicare Benefits Scheme (MBS) was heralded as a significant advance in supporting individuals with communication and swallowing difficulties (Medicare’s Chronic Disease Management Plan initiative (studies 8, 9, 23, 24, 29, 33, 39, 57, 71, 72), Medicare’s Helping Children with Autism and Better Start initiatives (studies 13, 19, 82). Since 2004, five MBS items have been legislated: (a) Enhanced Primary Care Plan initiative (name changed to Chronic Disease Management Plan) was introduced in 2004; (b) in 2008, Helping Children with Autism was added to the MBS; (c) in 2011, Better Start for Children with Disabilities was added to the MBS; (d) in 2023, Complex Neurodevelopmental Disorder & Eligible Disabilities was added to the MBS and superseded both Helping Children with Autism and Better Start for Children with Disabilities; and (e) in 2023, nasendoscopy or sinoscopy or fiberoptic examination of nasopharynx and larynx (unilateral or bilateral examination) was permitted by an eligible speech-language pathologist on behalf of a specialist of otolaryngology head and neck surgery [150]. These public funding provisions opened opportunities to access once-prohibitively expensive SLP services through private operators with the assistance of a partial rebate. This scoping review suggests that expanding subsidies will increase access to services by reducing the financial barriers to families. Figure 2 Supplementary Material V presents the total number of SLP services of four subsidised Medicare Benefit Schedule items and national population growth over five time points. This figure illustrates that Medicare’s Chronic Disease Management Plan (MBS_CDMP) had the greatest number of SLP services accessed in comparison to the other health insurance items. Medicare’s Chronic Disease Management Plan’s trajectory follows upward population growth across four time points (July 2004–June 2005, July 2008–June 2009, July 2013–June 2014, and July 2018–June 2019); however, there was a 44 % reduction between two time points (July 2018–June 2019 and July 2023–June 2024). It is important to note that the National Disability Insurance Scheme (NDIS) was introduced in 2013, with full scheme rollout in July 2020 [14]. As Figure 2 Supplementary Material V (Comparison of Medicare Benefits Schedule items for speech-language pathology and population growth) illustrates, the number of services for assessment and treatment items for Medicare’s Helping Children with Autism (MBS_HCWA) and Better Start for Children with Disability (MBS_BS) may reflect the diversity of public funding provisions available (i.e., the number of SLP services reported for MBS_HCWA and MBS_BS may be due to the availability of NDIS funding).

*Note: Refer to main article for references.*

Figure 2 Supplementary Material V

Comparison of Medicare Benefits Schedule items for speech-language pathology and population growth


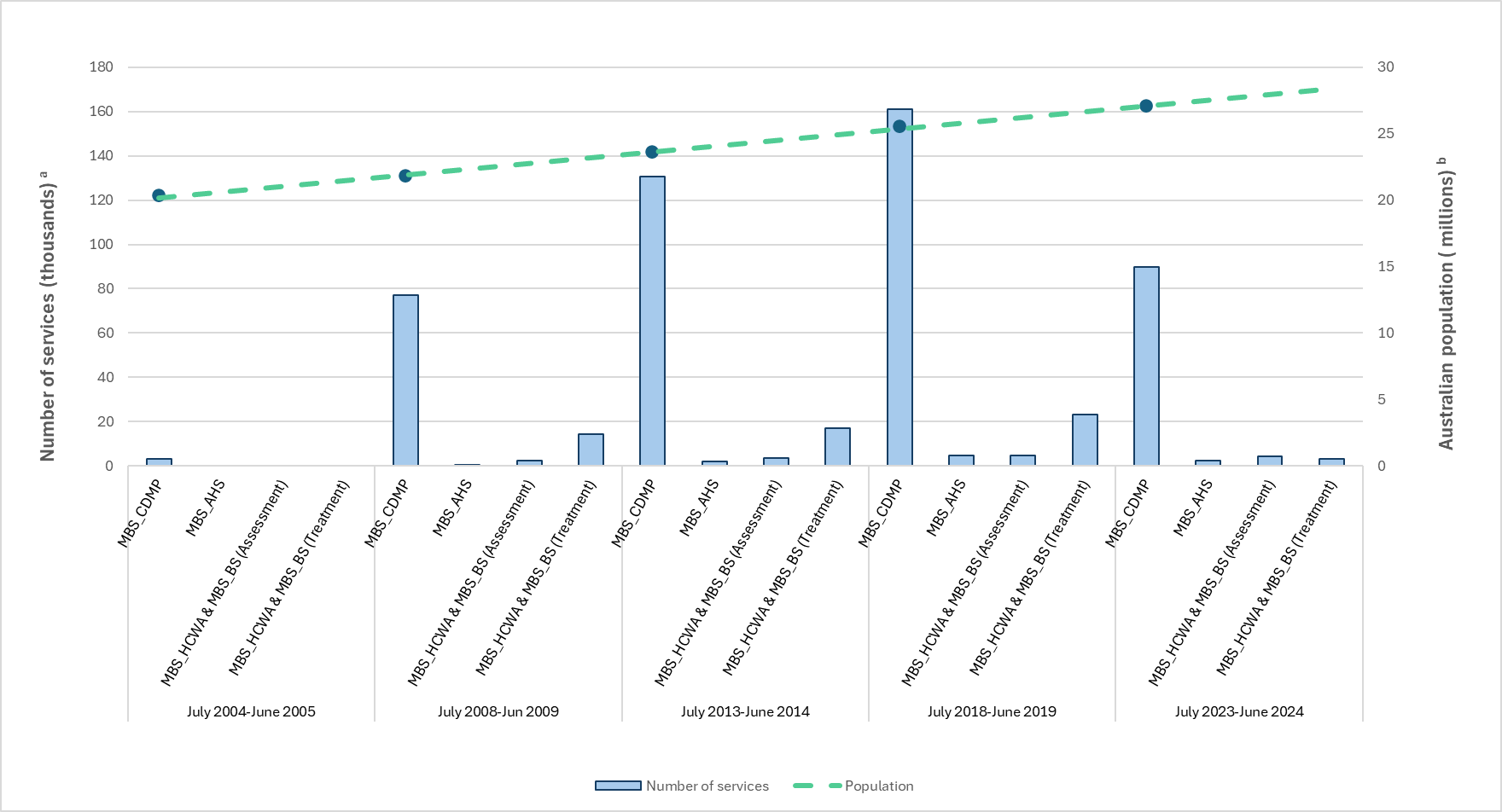


^d^

^c^

*Note. This figure compares four Medicare Benefits Schedule (MBS) items for speech-language pathology services across five time points (July 2004–June 2005; July 2008–June 2009; July 2013–June 2014; July 2018–June 2019; July 2023–June 2024). Data include all age groups. Australian population growth over the same periods is also presented. MBS_CDMP = Medicare Chronic Disease Management Plan; MBS_AHS = Medicare Allied Health Services for Aboriginal and Torres Strait Islander peoples; MBS_HCWA = Medicare Helping Children with Autism; MBS_BS = Medicare Better Start for Children with Disability.
^a^Data derived from Medicare item reports [13]. Available from:* [*http://medicarestatistics.humanservices.gov.au/statistics/mbs_item.jsp*](http://medicarestatistics.humanservices.gov.au/statistics/mbs_item.jsp) *[cited 2024 Oct 2].
^b^Population data derived from Australian Bureau of Statistics [164]. Available from:* [*https://www.abs.gov.au/statistics/people/population/national-state-and-territory-population/mar-2024*](https://www.abs.gov.au/statistics/people/population/national-state-and-territory-population/mar-2024) *[cited 2024 Sep 12]. ^c^As of July 1, 2004, only one MBS item was available (MBS_CDMP).
^d^As of March 1, 2023, MBS HCWA and MBS BS were superseded by the Complex Neurodevelopmental Disorder and Eligible Disabilities scheme. Item numbers remained unchanged (82005, 82020). References are provided in the main article.*
